# A latent invader: transcriptomics reveals *Cercospora zeina*’s stealth infection strategy of maize and immune-activating effectors

**DOI:** 10.3389/fpls.2025.1703682

**Published:** 2025-11-07

**Authors:** Trystan Nadasen, Carla Buitendag, Rodé Visser, Tanya Welgemoed, Ingo Hein, Dave Kenneth Berger

**Affiliations:** 1 Department of Plant and Soil Sciences, Forestry and Agricultural Biotechnology Institute (FABI), University of Pretoria, Pretoria, South Africa; 2 Potato Disease Resistance Group, James Hutton Institute, Dundee, Scotland, United Kingdom; 3 School of Life Sciences, University of Dundee, Dundee, United Kingdom

**Keywords:** RNA-seq, gray leaf spot, cell death, *Nicotiana*, Ecp2, NIS1a, GLS, necrotroph

## Abstract

*Cercospora zeina* is a fungal pathogen that causes gray leaf spot (GLS) disease on maize (*Zea mays* L.). Upon landing on a leaf, the pathogen enters through the stomata and continues to develop asymptomatically for up to 28 days before symptoms appear. As previous work has yet to adequately determine how the pathogen behaves during its infective period, we used transcriptomics to gain insights about the *in-planta* development of the pathogen and explore its use of effectors to facilitate this process. Samples from B73 maize inbreds inoculated with an African reference strain of *C. zeina* (CMW25467) were harvested in a time course experiment and used for deep RNA sequencing. We used reads mapped to an improved assembly and annotation of the *C. zeina* CMW25467 genome as a proxy for biomass accumulation. Following the latent period, *C. zeina* was found to rapidly accumulate biomass and showed a nearly 50-fold increase in biomass as symptoms appeared. Two distinct transcriptional waves occurred across the infection period. The first wave showed expression of genes for cellular growth, maintenance and immune avoidance, whereas the second wave was enriched with genes involved in detoxification and carbohydrate catabolism. A total of 140 putative effector genes were differentially expressed over the time course, with most upregulated during the mid stage when the switch to necrotrophy occurred. Transient expression of three of these *C. zeina* effectors (*CzEcp2*, *CzNIS1a*, *CzNIS1b*) induced plant immunity in *Nicotiana* spp. resulting in the development of cell death. The CzNIS1a effector required a signal peptide for activity in *Nicotiana benthamiana*, indicating that it is most likely secreted into the apoplast for this function. The previously undescribed CzNIS1b family member has an N-terminal domain with high sequence and structural identity to CzNIS1a plus a C-terminal domain made up of four alpha helices. Orthologues of CzNIS1b appear to be limited to the Mycosphaerellaceae. This study suggests that a cohort of *C. zeina* effectors expressed during the mid-stage of infection have functions for which receptors are present in non-host species like tobacco. Altogether, this work suggests *C. zeina* behaves as a latent necrotroph and provides a foundation for future research into the infection biology of *C. zeina.*

## Introduction

The Dothideomycete fungus *Cercospora zeina* causes gray leaf spot disease (GLS) on maize (*Zea mays* L.) (Crous et al., 2006; Meisel et al., 2009; Nsibo et al., 2019) in Africa, where maize is a staple food crucial to the food security of millions (Nsibo et al., 2024). GLS is characterized by the development of necrotic lesions on infected leaves (Berger et al., 2014; Nsibo et al., 2021) which reduce the photosynthetic capacity of the plant and severely limits the quality and quantity of harvests (Ward et al., 1999; Nsibo et al., 2021). Reallocation of resources from the stem and roots to compensate for the loss of photosynthates renders these tissues vulnerable to secondary infection with stem and root diseases resulting in premature lodging and further yield losses (Ward et al., 1999).

Infection typically occurs when wind or rain splash disseminated conidia land on the leaves of a susceptible maize genotype (Nsibo et al., 2021). Upon landing on a leaf, *C. zeina* conidia geminate and produce hyphae that grow towards and eventually penetrate the leaf through its stomata (Marais et al., 2024), mirroring observations made in the sister species *Cercospora zeae-*maydis (Beckman and Payne, 1982). Depending on environmental conditions and host susceptibility (Berger et al., 2014; Marais et al., 2024), the fungus can remain latent for up to 28 days before chlorotic leaf spots eventually occur (Ward et al., 1999; Crous et al., 2006) and develop into necrotic lesions (Korsman et al., 2012; Christie et al., 2017; Swart et al., 2017). Upon lesion development, fungal stroma form in the substomatal spaces after which conidiophores develop and emerge through the stomata (Caldwell and Laing, 2005), thereby completing the infection process.

Traditionally, phytopathogens have been classified into two broad groups based on their lifestyle within their hosts. Biotrophic fungi require living cells to grow; forming intricate structures called haustoria that facilitate effector secretion and nutrient acquisition to complete their lifecycle (Nowara et al., 2010; Li et al., 2021). These fungi rely on effectors to protect the pathogen and suppress plant immunity (Hemetsberger et al., 2012; Mueller et al., 2013), and achieve this, for example, by targeted inactivation of plant proteases (Mueller et al., 2013), blocking hormone signalling pathways (Djamei et al., 2011; Navarrete et al., 2022) and by inhibiting programmed cell death (Pennington et al., 2019; Li et al., 2021).

In contrast, necrotrophic fungi tend to kill host cells (Spoel et al., 2007; Shao et al., 2021) and do not require the production of structures like haustoria to facilitate their infection (Müller et al., 2024). Disease is typically mediated by the production of cell-wall degrading enzymes (CWDEs), cell death-inducing effectors and toxins that work in concert to kill host tissues (Müller et al., 2024). These pathogens must also be able to maintain their growth and development in the presence of plant defences and thus also produce defensive proteins and effectors that mitigate the effects thereof (Shao et al., 2021). These effectors typically suppress host defences by interfering with pathogen perception (Zhang et al., 2018; Chen et al., 2021), and inactivating proteases (Yang et al., 2018a) to prevent harm to the pathogen.

Hemibiotrophic fungi show characteristics of biotrophy and necrotrophy in their lifestyle (Liao et al., 2022). Generally, these fungi initially exhibit a biotrophic phase in which they penetrate host tissues and produce effectors that suppress and reprogramme host immunity (Münch et al., 2008) to establish an intimate association with the host during a period typically referred to as the “asymptomatic period” (Précigout et al., 2019). Following the asymptomatic period, unknown factors trigger a switch from the biotrophic phase to the necrotrophic phase (O’connell et al., 2012) in which secondary hyphae together with effectors and metabolites are produced that invade and kill host tissues (Perfect and Green, 2001; Münch et al., 2008).

Stricter definitions restrict hemibiotrophic fungi to those that produce haustoria-like infection structures during the biotrophic phase (O’connell et al., 2012; Qin et al., 2020). Common amongst members in the *Colletotrichum* and *Magnaporthe* genera, these haustoria-like structures are a type of modified biotrophic hyphae surrounded by a membrane layer that creates an interface between the host and pathogen (Qin et al., 2020) to facilitate nutrient flow and effector translocation (Irieda et al., 2014; Qin et al., 2020).

Fungal pathogens such as *Zymoseptoria tritici* that have a cryptic hemibiotrophic lifestyle do not produce these haustoria-like structures have thus been termed “latent necrotrophs”. This term encompasses pathogens with a long latent period in which limited growth and minimal symptom development is observed, followed by a distinctive switch to a necrotrophic phase associated with significantly higher growth rates (Sánchez-Vallet et al., 2015). The similar term “latent pathogen” has also been used to describe various taxa in the Botryosphaeriaceae (Paolinelli-Alfonso et al., 2016; Yan et al., 2017) and describes these pathogens as endophytes that become pathogenic, in response to unknown host/environmental cues (Yan et al., 2017); essentially describing the same phenomenon observed in Z. *tritici*.

Although fungal pathogens interact with their hosts in different ways, all fungi use effectors to facilitate their interaction (Toruño et al., 2016). Effectors are small molecules or proteins which are secreted into the host by the pathogen to alter the structure and function of the host to promote virulence (Du et al., 2014; Yin et al., 2019). These molecules are required for the pathogen to overcome plant defences and for it to complete its lifecycle in the host (Bos et al., 2006; Du et al., 2014; Dalio et al., 2018). Fungal effectors are typically less than 300 amino acids in length, although larger effector proteins have also been reported (Djamei et al., 2011; Presti et al., 2015; Mesarich et al., 2018). Fungal effector proteins can be split into two regions based on their structure. The N-terminal region typically contains the signal peptide, which is required for secretion of the mature effector protein from the pathogen into the host (Presti et al., 2015), as well as the translocation domain, which is required for the correct localisation of the effector into the host cell (Bos et al., 2006; Fang et al., 2017). The C-terminal region typically encodes the activity of the effector and is required for compatible/incompatible interactions to occur (Bos et al., 2006; Irieda et al., 2019).

Since its separation from *C. zeae-maydis* (Crous et al., 2006), most research on *C. zeina* has focused on host responses (Korsman et al., 2012; Berger et al., 2014; Christie et al., 2017; Meyer et al., 2017; He et al., 2021), evolution and pathogen diversity (Muller et al., 2016; Nsibo et al., 2019, 2021; Cheng et al., 2023; Welgemoed et al., 2023; Nsibo et al., 2024) with very little research focusing on its lifestyle and infection biology. Although some authors have suggested *C. zeina* behaves as a necrotrophic pathogen during infection (Benson et al., 2015), most work favours it having a more hemibiotrophic lifestyle (Meyer et al., 2017; Nsibo et al., 2021) even though almost nothing is known about how the pathogen develops during the latent period, and the haustoria-like structures typically associated with hemibiotrophs have not been observed in *C. zeina*.

Therefore, we hypothesize that *C. zeina* behaves more as a latent necrotroph than a hemibiotroph during infection and that effectors largely facilitate its switch to necrotrophy. To investigate this, a transcriptomics study was conducted to investigate gene expression trends and how the pathogen grows during the early, mid and late stages of disease. Candidate effectors were examined to identify effectors with shared expression profiles. Select *C. zeina* effectors expressed during the transition to necrotrophy were used in *Agrobacterium-*mediated transient transformation assays in a model system to investigate their potential roles during infection.

## Materials and methods

### Plant growth

Maize (*Zea mays*) plants of the B73 inbred line were grown in a PGW40 Conviron growth chamber (Conviron, Winnipeg, Canada). A transparent plastic barrier was installed in the Conviron to separate the inoculated and mock-inoculated plants. The chamber housed 40 single maize plants in plastic pots (20 cm diameter x 24 cm height). The growth chamber was controlled at the following conditions, which simulate a characteristic maize growing season and region (e.g. summer at Cedara Agricultural Research Station, Pietermaritzburg, South Africa: 28°C day/16°C night, up to 1400 µmol m^−2^s^−1^ photosynthetic photon flux density, 13-hour photoperiod, 60% relative humidity, which was increased to 100% (RHmax) for 2 weeks post inoculation).

A silica sand: Culterra^®^ seedling mix (Culterra, Nietgedacht, South Africa) (1:1 ratio (w/w)) was used as a plant growth medium. The plants were watered three times per day (7 am, 11 am and 4 pm) with 200–300 ml drip irrigated into the pots at each instance. Hygroponic fertilizer was used to fertilize the maize plants by gradually adding increasing concentrations of the water-soluble mixture every second day, starting with 1 g/L four days after emergence (dae) and incrementally moving up to 4 g/L by the silking stage. Maize growth and development were recorded once a week, whereby the leaf stage (V-stage) was counted using the leaf collar method (Purdue University, USA, https://extension.entm.purdue.edu/fieldcropsipm/corn-stages.php).

*Nicotiana benthamiana* and *Nicotiana tabacum* plants were grown from seed for 6 to 8 weeks. The seeds were germinated in peat jiffies (Grow-rite nursery supplies, Cape Town) for 2 weeks before being transferred to 10 cm pots containing a 1:1 ratio of river sand and compost for 4 to 6 weeks (Nadasen, 2022). The plants were grown in a phytotron under 16 hours of light (80 to 100 μmol m^-2^ s^-1^), at 25°C and 8 hours of darkness at 20°C per day. Relative humidity was maintained at 80% for the duration of the experiment. Plants were watered at regular intervals and fertilized with Nitrosol liquid fertilizer (Rural Research Limited) twice a week for the duration of the experiment.

### Maize inoculation trials

The B73 maize plants grown in the Conviron as described above were inoculated with the *C. zeina* CMW 25467 strain (Meisel et al., 2009) following the protocol adapted from Marais et al. (2024). In brief, prior to inoculation, defined sections (length = 10 cm, width across the whole leaf) of each leaf on all the plants were marked with a permanent marker pen. To prepare inoculum, *C. zeina* was cultured on V8 agar media in complete darkness at room temperature to promote conidiation. Conidia (renamed spores) were rinsed off using 0.01% Tween 20 and diluted in water to a concentration of 5x10^5^ spores/ml. The spore suspension was applied with the paintbrush method onto the abaxial and adaxial of the marked areas of the leaves of B73 maize plants at the VT stage of development. Mock-inoculated plants were treated with Tween 20 (0.01%) as a control for the experiment.

Four biological replicate maize leaf pieces per treatment were collected at three time points: 23, 30, and 44 days post inoculation (dpi). Replicates consisted of marked leaf areas on separate plants, with different plants sampled at each time point. Each replicate was photographed, immediately flash-frozen in liquid nitrogen, and stored at –80°C until further processing. Prior to freezing, the midveins of each leaf piece were excised and discarded since they lacked GLS lesions, and later - when frozen - were difficult to homogenize with a mortar and pestle at the first step of RNA extraction. Total lesion area was assessed using the leaf doctor application (Pethybridge and Nelson, 2015).

### *Cercospora zeina* genome assembly and annotation

An annotation for the Pacbio long-read sequence assembly (GCA_002844615.2) of *C. zeina* CMW 25467 described in Wingfield et al. (2022) was not available on GenBank at the start of the project. Therefore, we produced an updated assembly and annotation using the same Pacbio sequence reads. First, improvements were made to the GCA_002844615.2 genome assembly, and the update was submitted to GenBank as a new genome version, GCA_002844615.3. The new genome assembly included a round of polishing by Pilon (Walker et al., 2014) using the Illumina reads from the original draft genome assembly (GCA_002844615.1). The polishing method was described in the genome assembly reported by Wingfield et al. (2022), however the version submitted to Genbank (GCA_002844615.2) with that manuscript was mistakenly the version prior to the polishing. This was now corrected in the new assembly used in this study (GCA_002844615.3). In addition, two overlapping contigs were merged in the new assembly using the EMBOSS tool megamerger (Rice et al., 2000). Second, the automated annotation of the GCA_002844615.2 genome version described in Wingfield et al. (2022) was improved by transposing gene locations and splice sites from the previous published annotation (GCA_002844615.1) to the corresponding coordinates in the new genome, and curated by inspecting and correcting genes that failed GenBank’s validation. The gene models were assigned names using the UniRule (MacDougall et al., 2021) annotation system.

### Data and bioinformatic tools

The *C. zeina* CMW 25467 genome (Genbank Accession GCA_002844615.3) and the maize B73 reference genome (Genbank Accession GCA_902167145.1) (Jiao et al., 2017) were concatenated and used for read mapping in this study. EggNOG-mapper v2 was used for assignment of Kyoto Encyclopedia of Genes and Genomes (KEGG) terms as well as gene ontology terms (Cantalapiedra et al., 2021) to *C. zeina* genes. The proteome predicted for *C. zeina* CMW 25467 was analysed using SignalP6.0 (Teufel et al., 2022) for secretome prediction. Effectors were then predicted from the secretome using EffectorP 3.0-fungi (Sperschneider and Dodds, 2022). The localisation of candidate secreted effector proteins was predicted using SignalP6.0, EffectorP 3.0-fungi and localizer (Sperschneider et al., 2017).

### RNA isolations and RNA-sequencing

Total RNA was isolated from each sample using the QIAzol lysis reagent (Qiagen, Hilden, USA) followed by purification using the NucleoSpin RNA purification kit (Machery-Nagel, Duren, Germany). RNA quality was assessed using a NanoDrop 2000 spectrophotometer (Thermo Scientific, Waltham, USA), followed by agarose gel electrophoresis and RNA integrity analysis using a 2100 Bioanalyzer (Aligent, California, USA). For the RNA-seq, library preparation was done using a P3, PE100 kit (Illumina Inc. San Diego, USA) and sequenced using an Illumina NextSeq 2000 system at the Genomics Facility of the Core Facilities group at the James Hutton Institute.

### RNA-seq data analysis

An RNA-Seq data analysis workflow was built in Galaxy V24.1. Raw RNA-seq reads were assessed using FastQC to evaluate the quality of data, presence of adapter sequences and sequence characteristics such as read length and GC content. Trimmomatic (Bolger et al., 2014) was used to process the data to remove adapter sequences, low quality reads (AVGQUAL > 20) and short reads (MINLEN > 35). High quality reads were then mapped to the concatenated maize B73 reference genome (Genbank Accession GCA_902167145.1) (Jiao et al., 2017) and the *C. zeina* CMW 25467 genome (Genbank Accession GCA_002844615.3) using HiSAT2 (Kim et al., 2019) with default settings. The number of reads mapped to each gene was computed by htseq-count (Anders et al., 2015) using the union mode with a minimum alignment quality of 10. Data normalisation and differential expression analyses were computed using DEseq2 (Love et al., 2014) with a single factor (group) and three factor levels (early, mid and late). Pre-filtering (N > 38) was used to remove low expressed genes (outside 95^th^ percentile) from the dataset. A total of three contrast groups were set up to compare factor levels (late vs early, mid vs early and late vs mid) and differentially expressed genes (DEGs) were calculated for each contrast (Love et al., 2014).

### Analysis of differentially expressed genes, clustering and overrepresentation analyses

In this study, the expression pattern of a gene is defined as the collection of normalised read counts across all reps in the early, mid and late groups. Analysis of normalised read count data and analysis of differentially expressed genes (DEGs) was computed in R version 4.3.2. DEGs with a Log_2_ fold change (Log2FC) value > 1 or < -1 and an adjusted *p*-value (using the Benjamini-Hochberg procedure) < 0.05 were used for subsequent analyses unless otherwise stated. For heatmap clustering, normalized read counts were Z-score normalized, clustered by expression pattern and plotted using the Pheatmap R package. Gene clustering by expression pattern was performed by computing the dissimilarity (calculated using 1 - Pearson’s correlation coefficient) between each gene’s expression pattern followed by clustering using the Hclust R package. Overrepresentation analyses (ORAs) were computed using the clusterProfiler R package (Yu et al., 2012) to detect any KEGG and GO terms overrepresented in the dataset.

### Isolation of effector sequences from *C. zeina*

Total RNA was isolated from *C. zeina* mycelia grown on cornmeal agar as per standard protocols (Toni et al., 2018) before cDNA synthesis was performed using the Maxima H-minus first strand cDNA synthesis kit (Thermo Fisher Scientific, Massachusetts) using oligo (dT) primers followed by amplification using a polymerase chain reaction (PCR). Effector sequences were subsequently amplified using gene specific primers (**Supplementary Table S1**), cloned, and sequenced using DNA Sanger sequencing to verify the effector sequences.

### Vector construction

The *C. zeina Ecp2* (BST61_g11601) coding sequence (CDS) was isolated from *C. zeina* cDNA using the Ecp2 primer pair followed by PCR amplified using an Ecp2-specific primer set (**Supplementary Table S1**) to add *NcoI* and *BamH*I restriction enzyme recognition sites to the 5’ and 3’ ends of the *Ecp2* CDS respectively. The amplicon was then double digested using *NcoI* and *BamH*I (New England Biolabs, Massachusetts) as per standard protocols (Finney et al., 2001), electrophoresed and purified using the Zymoclean gel DNA recovery kit (ZYMO Research, California) before being ligated to the *NcoI* and *BamH*I double digested pTRAkc-ERH vector (Maclean et al., 2007) in a 3:1 ratio (insert to vector) using T4 ligase (New England biolabs, California) as per the manufacturer’s instructions.

The native signal peptide of CzNIS1a (BST61_g6509) was replaced with the *N. tabacum* PR1a signal peptide (Hammond-Kosack et al., 1994) using overhang extension PCR (OE-PCR) (**Supplementary Table S1**) (Makam et al., 2018; Hilgarth and Lanigan, 2020). Restriction enzyme recognition sites for *Age*I and *Xho*I were then added to the 5’ and 3’ ends of the gene using PCR (**Supplementary Table S1**). The amplicon was then double digested using *Age*I and *Xho*I (New England Biolabs, Massachusetts) as per standard protocols (Finney et al., 2001), electrophoresed and purified using the Zymoclean gel DNA recovery kit (ZYMO Research, California) before being ligated to the *Age*I and *Xho*I double digested pEAQ-HT vector, respectively (Leaf expression systems, Norwich) (Sainsbury et al., 2009) in a 3:1 ratio (insert to vector) using T4 ligase (New England biolabs, California) as per the manufacturer’s instructions. A variant of the *NIS1a* effector CDS lacking a signal peptide (NIS1a (No SP)) was similarly constructed using the pQmNIS1a primer (**Supplementary Table S1**) and inserted into the pEAQ-HT vector.

The tobacco PR1a signal peptide, FLAG epitope tag and CzNIS1a sequences were amplified using gene specific primers (**Supplementary Table S1**) to add *Bsa*I recognition sites to the 5’ and 3’ ends of each sequence. The sequences were then purified, mixed in a 5:1 ratio (insert: vector) with the pICH86988 vector (Guo et al., 2020) and added to a reaction with T4 ligase (New England biolabs, California), and the *Bsa*I restriction enzyme (New England biolabs, California) before being incubated (37°C for 1 minute followed by 16°C for 1 min) for 45 cycles to facilitate digestion and ligation of the sequences (Guo et al., 2020). The CzNIS1a (No SP) construct was similarly generated but used a forward primer (FmNIS1a-1F) to remove the PR1a signal peptide (**Supplementary Table S1**). The PR1a-FLAG-CzNIS1b gene was synthesized with flanking *Bsa*I recognition sites and ligated into a pUC58 plasmid (Eurofins genomics, Germany). A golden gate reaction was then set up as described above to create the pICH-PR1a-FLAG-CzNIS1b plasmid.

### Transformation of *Agrobacterium* and *Agrobacterium*-mediated transient transformation assays

The individual plasmids were transformed into electrocompetent *Agrobacterium tumefaciens* GV3101 cells containing the pMP90 helper plasmid by electroporation (Weigel and Glazebrook, 2006; Maclean et al., 2007) using an Eporator (Eppendorf, Hamburg). Transformed *Agrobacterium* cells were cultured separately in YEB media (no antibiotics) and incubated at 28°C for 3 hours with shaking (85 rpm) before being added to YEB agar media containing kanamycin (50 μg/ml) and gentamicin (25 μg/ml) (for strains with the pEAQ plasmid) or carbenicillin (50 μg/ml) and gentamicin (25 μg/ml) (for strains with the pTRAk plasmid). Liquid cultures were incubated at 28°C with shaking (85 rpm) until each culture reached an optical density measured at 600 nm (OD_600_) of 1. Each liquid culture was then centrifuged at 4000 rpm for 5 minutes (room temperature) before being resuspended in infiltration buffer (10mM MgCl_2_, 10mM MES and 100 μM acetosyringone, filter sterilized using a 0.22 μm syringe filter). The infiltration medium was left at room temperature in the dark for 3 hours to facilitate expression of the virulence genes.

Effector constructs were transiently expressed in the leaves of 6–8 week old *N. tabacum* (CzEcp2) or *N. benthamiana* (CzNIS1a and CzNIS1b) plants using the *Agrobacterium* mediated transient expression assay as per standard practices (Du et al., 2014). To reduce the effects of post-transcriptional gene silencing on gene expression, *Agrobacterium* suspensions were co-infiltrated with an *Agrobacterium* strain harbouring the pJL3-p19 plasmid in a 4:1 ratio (Lindbo, 2007). Leaves were photographed under visible light and/or ultraviolet (UV) light (365 nm) and the development of the cell death was monitored visually and photographed over a maximum period of 21 days.

### Protein extraction

Total soluble proteins were extracted from transiently transformed *N. benthamiana* leaves expressing the effector constructs. Leaf samples were harvested at 5 days post infiltration and frozen with liquid nitrogen before being ground into a fine powder. Two grams of each sample was then added to 2 ml of cold protein extraction buffer (10% glycerol, 25 mM Tris-HCl, 1 mM EDTA, 150 mM NaCl, 10 mM DTT and 0.1% tween 20) supplemented with a protease inhibitor cocktail mix (Sigma Aldrich, St. Louis). Samples were subsequently homogenized and incubated on ice for 20 minutes before being centrifuged at 15,000 x g. The pelleted plant debris was discarded and the remaining supernatant was used in a Bradford assay to quantify the concentration of each protein sample (Ernst and Zor, 2010).

### Western blotting

Crude protein extracts were separated using sodium dodecyl sulphate polyacrylamide gel electrophoresis (SDS-PAGE). The PAGE gel was electroblotted to a nitrocellulose membrane using a transfer buffer (20 mM Tris–HCI, 150 mM glycine and 20% methanol) before being rinsed with Tris-buffered saline (TBS). The membrane was subsequently blocked using a 1.5% casein (Sigma Aldrich, St. Louis) blocking buffer. Monoclonal ANTI-FLAG M2, Clone M2 primary antibodies (Sigma Aldrich, St. Louis) were prepared by dissolving the antibody in a blocking buffer as per manufacturer instructions before incubating the membrane in the antibody solution (1:1000 dilution). The membrane was washed with TBS before being incubated in solution of anti-Mouse IgG-Peroxidase (Sigma Aldrich, St. Louis) as per manufacture instructions (1:30000 dilution). Flag-tagged proteins were then detected by exposing the membrane to 3,3’5,5’-tetramethylbenzidine (TMB) and monitoring the membrane for the development of blue bands.

## Results

### Improved *C. zeina* genome assembly and annotation

The *C. zeina* CMW25467 genome was made more contiguous than the previous version by merging two overlapping contigs (Czeina_00010F and Czeina_00005F) from the GCA_022702355.2 assembly (Wingfield et al., 2022) into one larger contig (CZEINA_10_05) in the improved assembly GCA_022702355.3. The new merged contig was supported by its clear alignment with a single contig in a different *C. zeina* strain, namely the JAKLWX010000010 contig in the GCA_022702355.1 genome (Cheng et al., 2023). This resulted in a small decrease of 7 Kb from the merged overlap and one less contig, resulting in a 41.8 Mb assembly consisting of 21 contigs. This assembly was also improved by polishing the PacBio assembly with Illumina reads that had been generated in the first draft assembly (Wingfield et al., 2017). The read depth of the Illumina reads enabled higher accuracy and correction of some nucleotides in the Pacbio assembly, which was a limitation of this technology at the time.

The curated annotation of the improved *C. zeina* CMW25467 assembly (GCA_022702355.3) resulted in a total of 11,604 predicted protein coding genes, which was an increase of 34 compared to the GCA_002844615.2 annotation (Wingfield et al., 2022). The increase was attributable to the incorporation of previously unannotated genes and the resolution of certain gene models into two genes.

### Gray leaf spot disease symptoms and *C. zeina* RNA-seq read counts increase exponentially after the latent period

**Figure 1:**
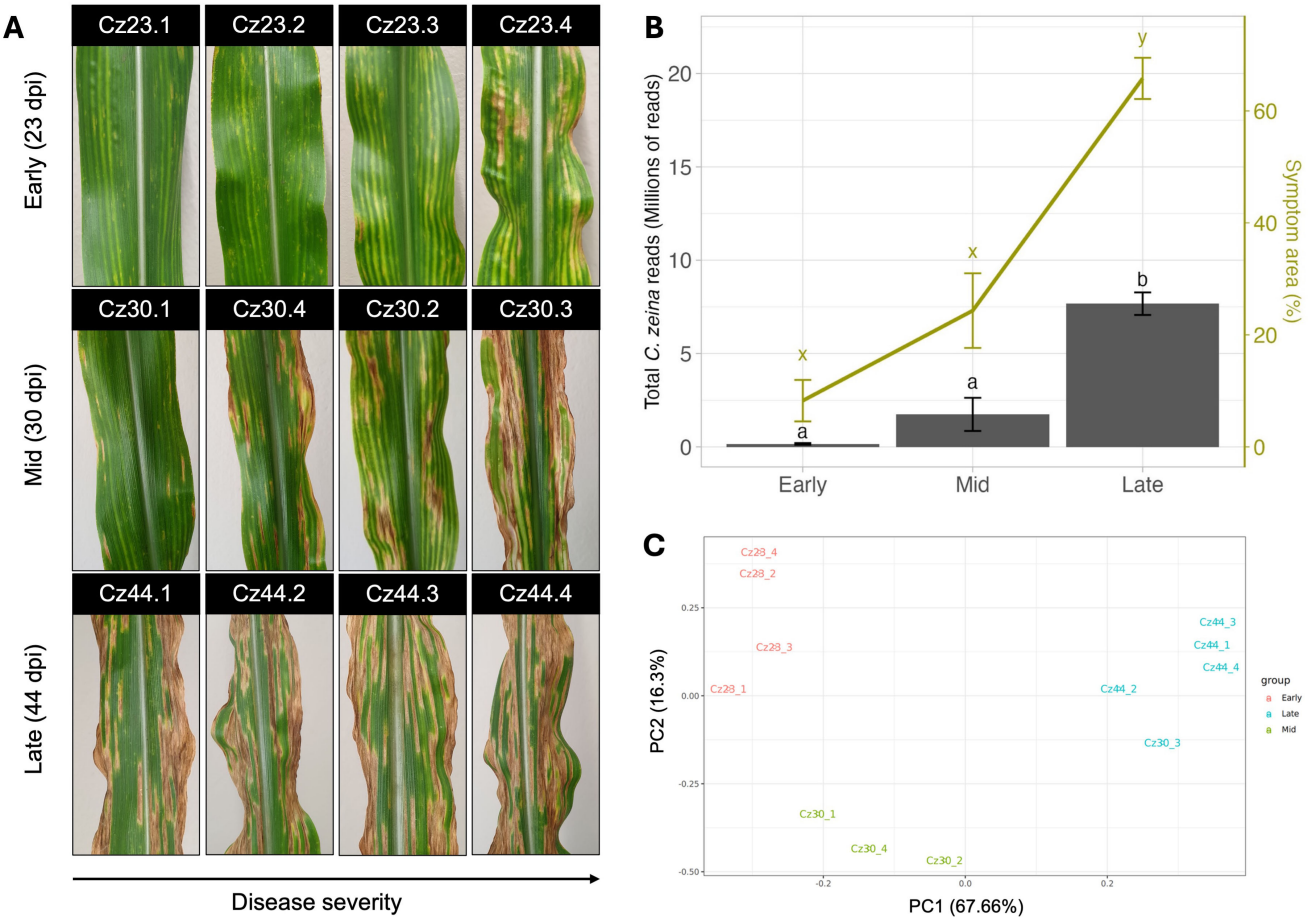
B73 maize leaves with symptoms of gray leaf spot disease for RNA sequencing. **(A)** Leaves were inoculated with *Cercospora zeina* conidia and harvested at 23-, 30- and 44-days post inoculation (dpi) representing the early, mid and late stages of infection, respectively. Harvested samples were photographed immediately following harvesting. Image credits: Kevin Scheepers. **(B)** Analysis of symptom area and RNA sequencing read count data from B73 maize leaves with symptoms of gray leaf spot disease. Leaves were inoculated with *Cercospora zeina* conidia and harvested at 23-, 30- and 44-days post inoculation (dpi) representing the early, mid and late stages of infection, respectively. The bar plot represents the total average reads mapped to the *Cercospora zeina* genome in the early, mid and stages of disease (left axis, black), whereas the area of the inoculated leaf showing symptoms in the early, mid and stages of disease is represented by the line graph (right axis, green). Error bars represent standard error, and treatments with the same letter are not significantly different from one another (α = 0.05). Model significance was tested using an ANOVA followed by a Tukey *post hoc* test to test for significance between factor levels. **(C)** Principal component analysis of RNA sequencing reads mapping exclusively to the *Cercospora zeina* genome in each of the 12 replicates following normalization by DEseq2. Replicates are coloured based on the point of infection at which the sample was harvested.

Leaves of the maize inbred line B73 were inoculated with *C. zeina* conidia and monitored for the development of GLS symptoms. A latent period was observed with chlorotic spots detected as the first symptom to develop at 23 days post infection (dpi) (**Figure 1A**). Further development of chlorotic spots across the time course resulted in the formation of rectangular and tan matchstick-like, yellow-bordered lesions within leaf veins that are typical of GLS (**Figure 1A**, 30 dpi). These eventually coalesced, causing significant damage to the infected areas of the leaves (**Figure 1A**, 44 dpi). We classified the samples collected at 23, 30 and 44 dpi as “early”, “mid” and “late” stages of infection, respectively. This was because the early stage was at the end of the latent period when the first symptoms appeared, the mid stage was at the appearance of typical GLS lesions, and the late stage was when most of the GLS lesions had expanded sufficiently to merge with other lesions (**Figure 1A**). Each inoculated area on all four biological replicate leaves per time point was photographed and scored to quantify the total average symptom area at each stage of infection. No disease symptoms were observed on the leaves of mock inoculated plants. The average symptom area of samples from the late stage of disease (44 dpi) were found to be significantly greater (*p* < 0.05) than the average symptom area observed in samples from the early (23 dpi) and mid-stages (30 dpi) of disease respectively, highlighting the rapid progression of infection within infected samples between the early and late stages of disease (**Figure 1B**).

Total RNA isolated from the infected samples was used for RNA-seq to examine changes in the *C. zeina* transcriptome across infection. Sequencing depth ranged between 67 and 92 million reads per sample, with mean quality scores (Phred scores) above 32 for all sequenced samples. Following quality control, filtering and mapping, the RNA-seq reads aligned to the *C. zeina* and *Z. mays* genomes were counted and evaluated. As expected, the number of reads mapped to the *Z. mays* genome remained relatively consistent between samples, with an average of 86% of reads in each sample mapping to the *Z. mays* genome (**Supplementary Table S2**; **Supplementary Figure S1**). The *Z. mays* and *C. zeina* transcript datasets were analysed separately to avoid bias, since the substantially larger maize transcriptome (>50,000 transcripts) could otherwise overshadow patterns in the comparatively smaller *C. zeina* transcriptome (<12,000 transcripts). Therefore, transcript data from *C. zeina* genes only were used for subsequent analyses. The average number of reads mapping to the *C. zeina* genome in each stage of disease was used as a proxy for *C. zeina* biomass (Blanco-Ulate et al., 2014; Lanver et al., 2018; Human et al., 2020). The mean number of reads mapping to the *C. zeina* genome at each stage increased exponentially across infection, with a more than 40 fold increase in mean read counts observed between the late and early stages of infection (**Figure 1B**; **Supplementary Table S2**). Read count and symptom area datasets were found to be strongly correlated (r = 0.98), indicating a link between GLS severity and fungal load (**Figure 1B**).

### Transcriptome analysis reveals two waves of *C. zeina* gene expression during maize infection

Prior to differential expression analysis, the Cz30.3 sample was observed to have the second highest number of *C. zeina* reads (10.56%) and GLS symptoms (73%) of all the samples (**Supplementary Table S2**), indicating that disease had progressed more rapidly in this sample (**Figure 1A**). This reflects the challenge of synchronizing disease progression when using the paintbrush method of leaf inoculation with fungal conidia, especially in fungi such as *C. zeina* which have a long latent period (Meisel et al., 2009). On account of the advanced disease development in the Cz30.3 sample, we grouped this sample with the other 44 dpi samples for the differential expression analyses. Following this, principal component analysis (PCA) was performed on the *C. zeina* reads in each biological replicate of each time point (**Figure 1C**). The PCA showed three clusters that corresponded to the early (samples 23.1 – 23.4), mid (samples 30.1, 30.2 and 30.4) and late (samples 44.1 – 44.4 plus 30.3) stages of disease, with the first two components explaining over 83% of the variation in the dataset (**Figure 1C**).

**Figure 2:**
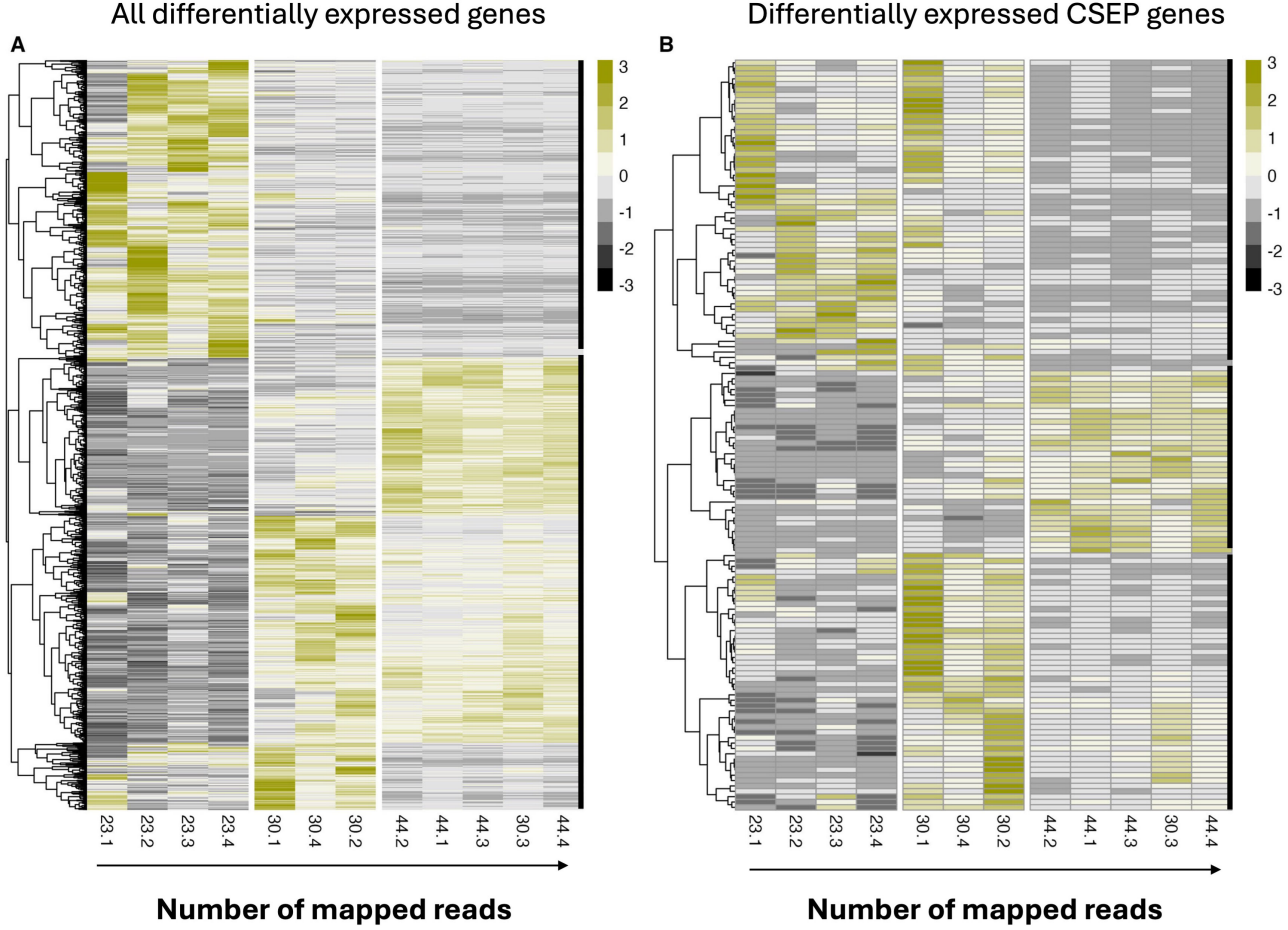
Heatmap of differentially expressed *Cercospora zeina* genes. **(A)** Normalized read counts were scaled and clustered by expression profile (row) using the complete clustering method. Replicates (columns) were arranged in order of number of reads mapped to the *C*. *zeina* genome. **(B)** Heatmap of differentially expressed *Cercospora zeina* candidate secreted effector protein (CSEP) genes. Normalized read counts were scaled and clustered by expression profile (row) using the complete clustering method. Replicates (columns) were arranged in order of number of reads mapped to the *C*. *zeina* genome. Thick black lines on the right of each heatmap represent clusters denoted for the analysis.

Analysis of the *C. zeina* transcriptome revealed the presence of 2986 genes that were differentially expressed in at least one of the three contrasts (**Figure 2A**) and included 140 genes encoding putative effector proteins (**Figure 2B**). **Table 1** shows that the largest number of differentially expressed genes (DEGs) was detected in the late-early contrast, followed by the mid-early and late-mid contrasts, respectively (**Table 1**). The late-early contrast also showed the highest number of differentially expressed genes in the experiment, highlighting the substantial change in gene expression that occurs between these two stages. Interestingly, the late-mid contrast showed the fewest DEGs, with less than a third of the DEGs observed in the late-early contrast. Heatmap clustering of DEGs exemplified these differences, and revealed the presence of two broad gene clusters with distinct expression patterns (**Figure 2A**); one of which showed peak expression in the early stage and the other which showed peak expression in the mid and/or late stage of infection. Altogether, this information suggests that two distinct waves of pathogen gene expression occur during infection.

**Table 1:** Differentially expressed *Cercospora zeina* genes (DEGs) compared between the early, mid and late stage of disease on maize.

Contrast	Upregulated DEGs^1^	Downregulated DEGs	Total DEGs
Late vs Early	1200	1029	2229
Late vs Mid	250	415	665
Mid vs Early	1122	761	1883

^1^Higher expression in the treatment listed first in the “contrast” column.

### Contrasting processes are upregulated in the first and second waves of expression

**Figure 3:**
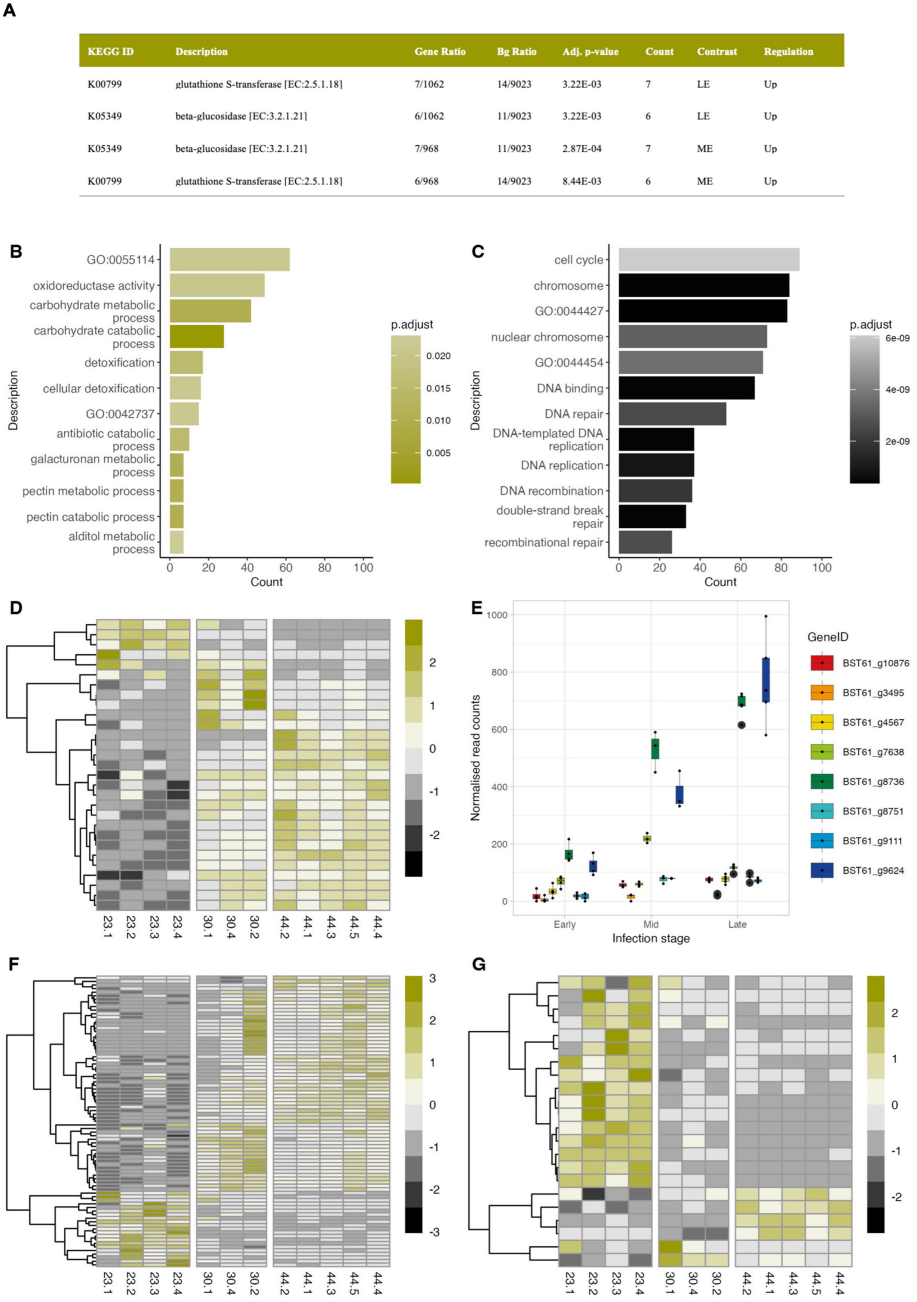
Analysis of differentially expressed *Cercospora zeina* genes during its infection of maize. **(A)** Overrepresentation analysis (ORA) of Kyoto Encyclopaedia of Genes and Genomes (KEGG) ortholog terms identified in the upregulated genes during *Cercospora zeina* infection of maize showing significantly overrepresented pathways (α = 0.05) in the late-early (LE), mid-early (ME) and late-mid (LM) contrasts. **(B)** Results from the Gene Ontology (GO) ORA showing the 12 most significantly overrepresented GO terms (α = 0.05) amongst the differentially expressed upregulated genes in the late-early contrast. GO:0042737 is a secondary ID for GO:0042178 (xenobiotic catabolic process) and GO:00555114 represents the GO term name for “oxidation-reduction process”. **(C)** Results from the Gene Ontology (GO) ORA showing the 8 most significantly overrepresented GO terms (α = 0.05) amongst the differentially expressed downregulated genes in the late-early contrast. GO:0044427 represents the GO term name “chromosomal part”; GO:0044454 represents GO term name “nuclear chromosome” **(D)** Genes mapped to the gene ontology term GO:0009636 (response to toxic substance) that are differentially expressed in at least one contrast. **(E)** Boxplots showing upregulated differentially expressed genes involved in the glutathione-s-transferase pathway in the LE and ME contrasts. **(F)** Heatmap showing the expression of differentially expressed glycoside hydrolase genes across infection. **(G)** Heatmap showing the expression of differentially expressed glycosyltransferase genes across infection.

Pathogen metabolic pathways and gene ontologies were investigated using KEGG and GO overrepresentation analyses (ORAs) (Yu et al., 2012). KEGG ORAs showed that upregulated genes in the late-early and mid-early contrasts had an overrepresentation of enzymatic pathways involving glutathione-s-transferases (GST) (KEGG ID: 00799) and beta-glucosidases (KEGG ID: K05349) (**Figure 3A**). GO ORAs complimented these results and showed that upregulated genes in the mid and late stages had an overrepresentation of GO terms associated with detoxification, carbohydrate metabolism and secondary metabolism (**Figure 3B**; **Supplementary File 1**). Interestingly, gene ontologies involving the cell cycle, DNA replication and DNA binding were expressed in the early stage and significantly downregulated thereafter (**Figure 3C**). Significantly overrepresented GO terms were not detected amongst up- and downregulated genes from the late-mid contrast groups, highlighting the overlap in gene expression between these two stages.

Detoxification-related processes overrepresented in both KEGG and GO ORAs were examined further. A total of 31 differentially expressed genes were mapped to the “response to toxic stress” (GO:0009636) gene ontology which encompasses the detoxification (GO:0098754) and response to toxic substance (GO:0097237) GO terms (**Figure 3D**). Almost all of these genes (n = 27) showed relatively low expression levels during the early stages of disease but were significantly upregulated in the mid and late stages (**Figure 3D**), indicating detoxification-related processes are largely induced during the mid and late stages of disease. Investigation of genes involved in GST pathways exemplified these trends and highlighted two genes (BST61_g9624 and BST61_g8736) that showed a 4-fold increase in expression between the late and early stages (**Figure 3E**).

Beta-glycosidase pathways and gene ontologies related to carbohydrate metabolism were similarly overrepresented amongst upregulated genes in the mid and late stages, suggesting carbohydrate processing is favoured during these stages. Investigation of differentially expressed carbohydrate-active enzymes (CAZymes) showed glycoside hydrolases (GH) to be the most represented CAZyme class. A total of 81 GH genes showed differential expression, of which 60 genes showed peak expression in the mid and/or late stages of disease (**Figure 3F**; **Supplementary Table S3**). The vast majority of these genes were found to belong to CAZyme families that hydrolyse components of cellulose and hemicellulose (Xylan) (**Supplementary Table S3**). A limited number of GH genes (n = 21) showed peak expression in the early phase and were subsequently downregulated in the mid and/or late stages. Over a third of these genes belonged to GH families involved in fungal cell wall synthesis (GH 16, 17, 18, and 76), whereas the rest belonged to GH families involved in plant cell wall degradation (GH 3,5, 10, 55 and 88).

Pectin metabolism was significantly overrepresented in the GO ORAs of upregulated genes in the mid-early and late-early contrasts (**Figure 3B**). A total of sixteen genes with roles in pectin degradation were identified throughout the infection. Mid to late stage expression was the dominant expression pattern observed, with ten of the twelve differentially expressed genes showing significant upregulation in the mid and/or late stages of disease (**Supplementary Figure S2**). Notably, BST61_g5290, a gene encoding a putative pectate lyase with high structural similarity to the VdPEL1 virulence factor from *Verticillium dahliae* (Yang et al., 2018b) (**Supplementary Figure S2**) was one of the most highly expressed genes (99.5^th^ percentile) in the late stage dataset. Altogether, this information suggests pectin degradation is an important process during infection.

Glycosyltransferases (GT) were the next most well represented class of CAZymes with twenty one differentially expressed GT genes identified in the experiment (**Figure 3G**). In contrast to the *C. zeina* GHs, only a few GT genes (n = 6) were significantly upregulated in the mid or late stage; most of which belonged to GT families involved in the synthesis of cell wall components like chitin (**Supplementary Table S3**). Interestingly, the majority of GTs were expressed in the early stage of infection and significantly downregulated thereafter. Half of these genes were grouped into the GT25 and GT31 families, both which are likely to play roles in fungal growth and developmental processes such as cell wall synthesis and post-translational modification of proteins (**Supplementary Table S3**).

The expression pattern of proteins known to interact with components of the fungal cell wall were investigated across the experiment. A total of six hydrophobin genes were identified in the *C. zeina* genome (**Supplementary Figure S3**). All six hydrophobin genes contained signal peptides at their N-terminals and were predicted as effectors by EffectorP3.0-fungi. These proteins also contained eight or more conserved cysteine residues and were classified as class I or class II hydrophobins based on their primary structure. BST61_g6910 and BST_g1984 were significantly upregulated in the mid-early and late-early contrasts and fell within in the 99.8^th^ percentile of average expression in the late stage, representing the 6^th^ and 9^th^ most highly expressed genes in the late stage, respectively (**Supplementary Figure S3**). In contrast, the remaining four hydrophobins showed high expression in the early phase, but were significantly downregulated thereafter leading to an approximately seven fold decrease in gene expression in all four genes by the late stage. No relationship between class I and class II hydrophobins in terms of gene expression was detected.

Homologs of the chitin-binding extracellular protein 6 (Ecp6) and avirulence protein 4 (Avr4) genes are represented by the BST61_g10156 (CzEcp6) and BST61_g2073 (CzAvr4) genes, respectively. Both genes showed higher expression in the early and mid-stages of disease compared to the late stage (**Supplementary Figure S4**). BST61_g10156 was one of the most highly expressed genes across the experiment, falling into the 98^th^ percentile of expression (or higher) in all stages of infection, but was not differentially expressed (**Supplementary Figure S4**). BST61_g2073 was expressed to a lower extent throughout the experiment and was also constitutively expressed (**Supplementary Figure S4**). Interestingly, homologs of Avr4 in related *Cercospora* species have also been shown to regulate the synthesis of the toxin cercosporin (Rezende et al., 2019). Despite the lack of production of cercosporin by *C. zeina* (Swart et al., 2017), the genes in the cercosporin toxin biosynthesis (CTB) pathway were previously shown to be expressed throughout infection (Swart et al., 2017). Data from this experiment supported that observation and showed all of the *CTB* genes to be expressed during infection (**Supplementary Figure S4**), with most of these genes showing increased expression in the mid stage of disease.

Comparison of expression data from conviron and field experiments showed similar *CTB* gene expression trends between the two experiments. Correlation coefficients calculated between the conviron data (early, mid and late groups) and the field data (**Supplementary Figure S4**) showed that the conviron dataset was strongly correlated to the field dataset, and that data from the mid stage of disease from the conviron experiment showed a near perfect correlation (r = 0.94) to the field dataset (**Supplementary Figure S4**). Comparisons made using *CTB* gene expression data from *C. zeina* grown *in vitro* on cornmeal, V8 agar, complete media or yeast extract, peptone and dextrose (YPD) agar (Swart et al., 2017) further showed that the conviron data was best correlated to *in vitro* expression data from culture media that mimicked natural conditions (cornmeal and V8), whereas artificial media like complete and YPD media were correlated to a lower extent (**Supplementary Figure S4**). Altogether, this data shows that the CTB cluster including the truncated *CTB7* gene is expressed despite the absence of cercosporin and suggests that the pathway may have alternative biosynthetic functions.

### The majority of *C. zeina* candidate secreted effector proteins are upregulated during the switch to necrotrophy

Effector proteins are responsible for promoting a pathogen’s proliferation and can provide clues towards understanding how the pathogen and host interact at the molecular level during an infection. A total of 953 secreted proteins were predicted from a pool of 11,604 predicted proteins in the *C. zeina* genome, with nearly one third of the secreted proteins classified as candidate secreted effector protein (CSEP) (**Table 2**). A total of 140 CSEP genes were differentially expressed in at least one of the three contrasts, with the majority of the differentially expressed CSEPs predicted to have apoplastic localisation (**Supplementary Table S4**). Several CSEPs with cytoplasmic localisation were also identified, along with 28 CSEPs identified to contain nuclear localisation signals (NLS) of which 7 were differentially expressed.

**Table 2:** Predicted proteins in the *Cercospora zeina* genome.

Proteins	Total	^1^Differentially expressed
All proteins	11604	2986
Secreted proteins	953	421
^2^CSEPs	312	140

^1^Protein products of genes showing differential expression in at least one contrast.

^2^CSEP - candidate secreted effector protein.

**Figure 4:**
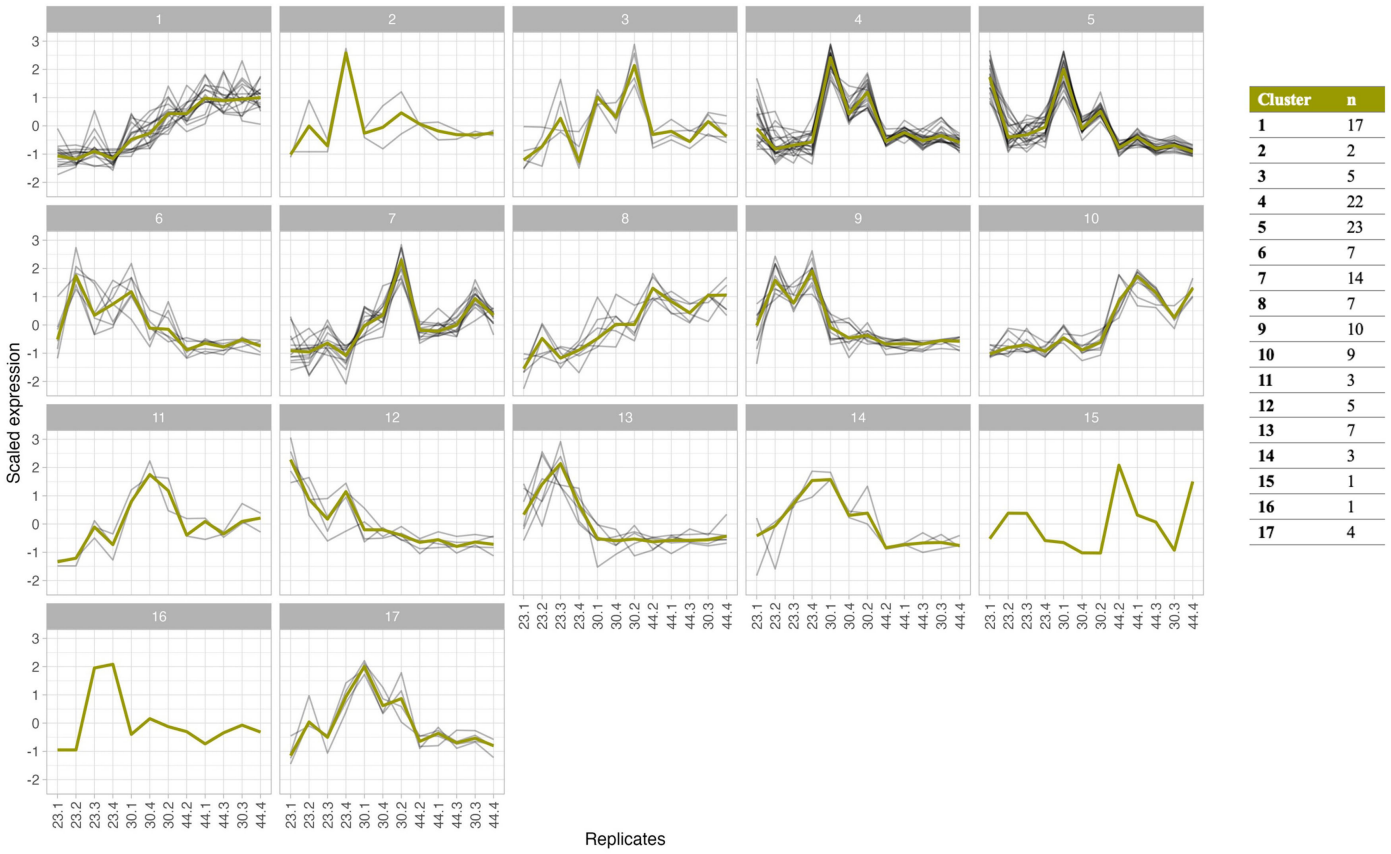
Co-expression clustering analysis of 140 differentially expressed *Cercospora zeina* candidate secreted effector protein (CSEP) genes. The expression pattern of each was scaled for the purpose of comparison. The Pearson correlation coefficient was used to determine the dissimilarity (1 - correlation coefficient) between the expression profiles of each gene. Clustering was computed using the complete clustering method. Clusters with a dissimilarity score less than 0.5 (Moderately positively correlated) are shown. The thick green line in each cluster represents the mean expression profile for each cluster.

Analysis of the expression patterns of the differentially expressed CSEPs highlighted three broad groups of CSEPs expressed in the early, mid and late stages of disease (**Figure 2B**). Further clustering analyses grouped these CSEPs into seventeen distinct clusters, with each cluster showing a moderate to strong positive Pearson correlation (r > 0.5) across the time course (**Figure 4**). The majority of clusters showed peak expression in the mid and late stages of infection, with relatively few clusters showing peak expression in the early stage. Interestingly, most of the differentially expressed CSEPs were represented in only four clusters (clusters 1, 4, 5 and 7), all of which show peak expression in the mid and/or late stages of disease development (**Figure 4**).

**Figure 5:**
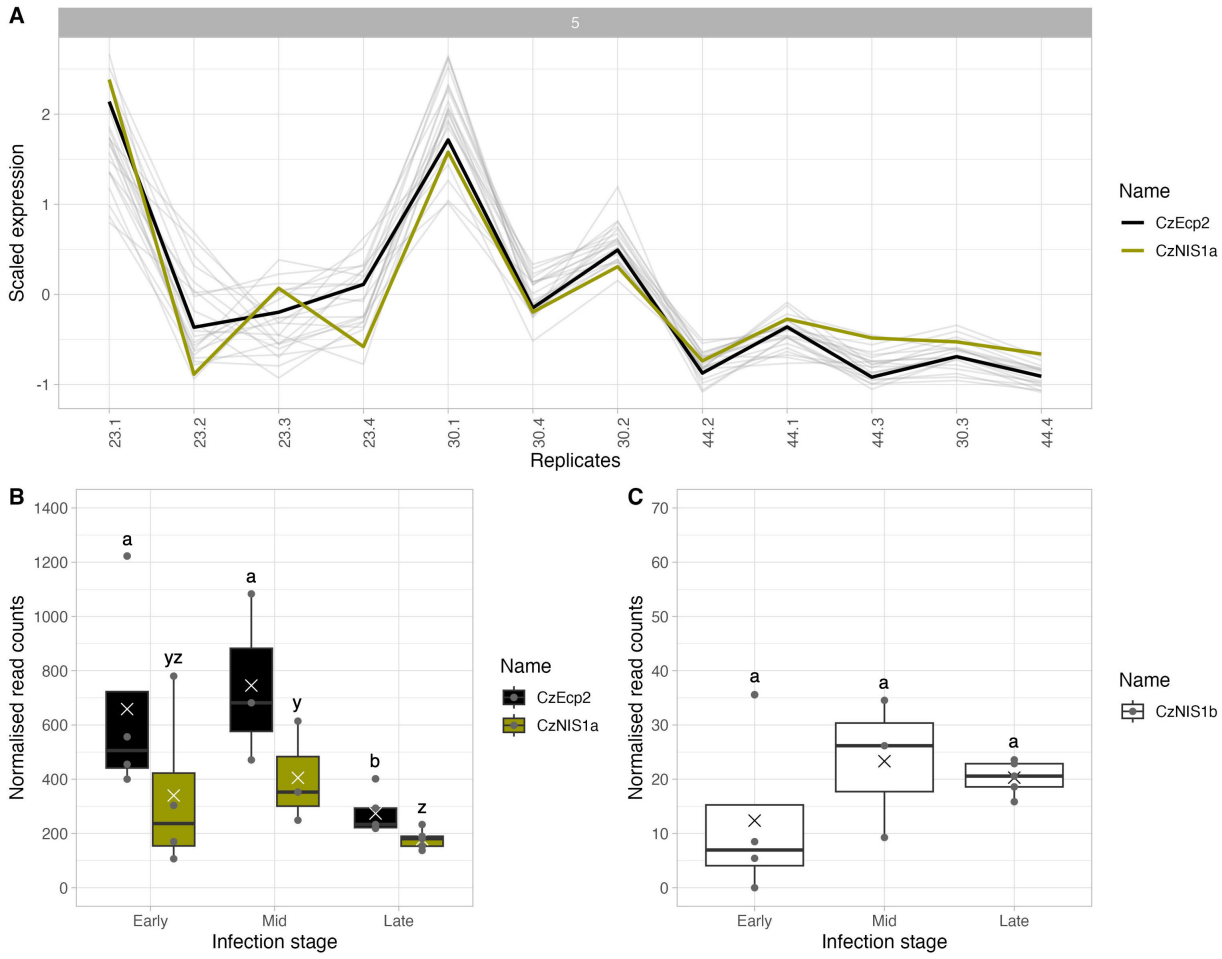
Expression patterns for candidate secreted effector proteins (CSEPs) from *Cercospora zeina* during the early, mid and late stages of GLS infection on B73 maize inbreds. **(A)** Co-expression clustering analysis of 23 differentially expressed *Cercospora zeina* candidate secreted effector protein (CSEP) genes in cluster 5. The expression pattern of each gene was scaled for the purpose of comparison. The Pearson correlation coefficient was used to determine the dissimilarity (1 - correlation coefficient) between the expression profiles of each gene. Clustering was computed using the complete clustering method. The expression patterns of the *C*. *zeina* necrosis inducing secreted protein 1a (CzNIS1a) and Extracellular protein 2 (CzEcp2) CSEPs are shown in green and black, respectively). **(B)** Normalised read counts for the *C*. *zeina* necrosis-inducing secreted protein 1a (CzNIS1a) and Extracellular protein 2 (CzEcp2) CSEPs. **(C)** Normalised read counts for the *C*. *zeina* necrosis-inducing secreted protein 1b (CzNIS1b). Genes that are not significantly differentially expressed (adjusted *p*-value < 0.05 and log2FC > 1 or log2FC < -1) in the Late-Early, Mid-Early and/or Late-Mid contrasts are represented by the same letter.

Peak expression during the mid stage of infection was the dominant pattern, with more than half of the differentially expressed CSEPs (n = 74) reaching maximum expression at this time. Cluster 5 was the largest cluster of differentially expressed CSEPs to show this expression pattern and contains 23 members with closely related expression patterns (r > 0.66) (**Figure 5A**). Gene expression in the cluster peaked in the mid stage, after which expression decreased sharply in the late stage (**Figure 5A**).

### Structure/function predictions of *C. zeina* candidate secreted effector proteins

**Figure 6:**
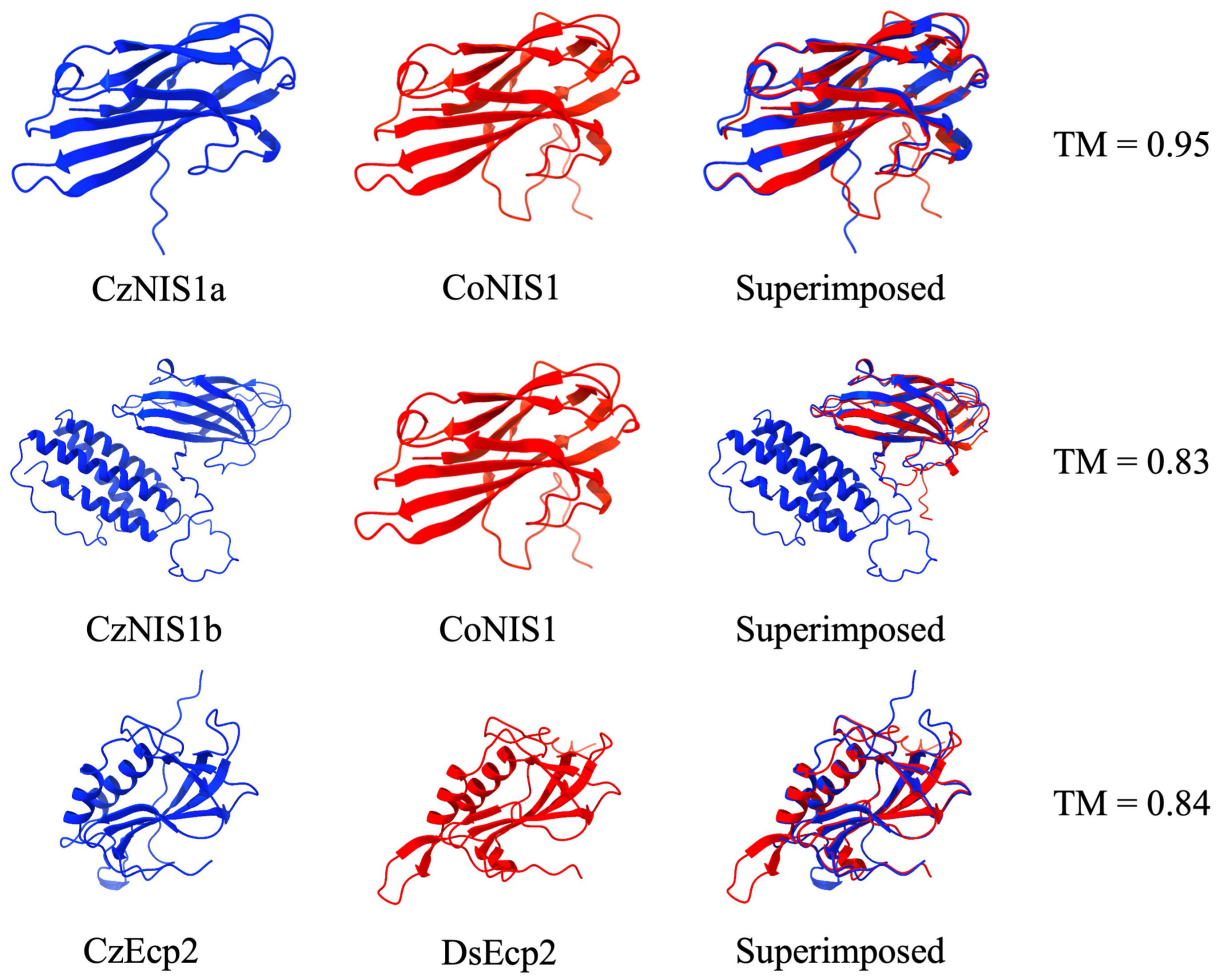
AlphaFold3 models of the *Cercospora zeina* necrosis-inducing secreted protein 1a (CzNIS1a), *C. zeina* necrosis-inducing secreted protein 1b (CzNIS1b) and the *C.zeina* extracellular protein 2 (CzEcp2) candidate secreted effector proteins (CSEPs). Necrosis-inducing homologs from *Colletotrichum orbiculare* (CoNIS1) and *Dothistroma septosporum* (DsEcp2) were similarly modelled, superimposed and compared to each of the respective *C. zeina* CSEPs using TM-align to generate a template modelling scores (TM) for each comparison.

BLASTp analysis of genes in cluster 5 revealed the presence of *C. zeina* homologs of the necrosis inducing secreted protein 1 (NIS1) (Irieda et al., 2019) and extracellular protein 2 (Ecp2) effectors (Stergiopoulos et al., 2010), both of which were similarly expressed during infection (**Figure 5B**). The *C. zeina* NIS1 homolog (BST61_g6509, designated CzNIS1a) is encoded by a 570 bp gene, contains two exons encoding a 149 amino acid protein with a signal peptide (18 amino acids) predicted by SignalP6.0 at the N-terminus (probability = 0.997) and is predicted as an apoplastic effector by EffectorP 3.0-fungi. Cysteine residues, conserved protein domains and subcellular localisation signals were absent from the predicted CzNIS1a protein. Alphafold3 modelling of CzNIS1a and the CoNIS1 effector homolog (Yoshino et al., 2012) from *Colletotrichum orbiculare* revealed the two structures to be remarkably similar (TM-score = 0.95), possibly hinting at them having similar functions (**Figure 6**).

Subsequent BLASTp analyses using CoNIS1 as a query revealed a second, much larger homolog of NIS1 in the *C. zeina* genome and was thus designated as CzNIS1b (BST61_g6426). The CzNIS1b gene is encoded by a 1,134 bp gene and contains 5 exons, encoding a 299 amino acid protein including a signal peptide (17 amino acids) predicted by SignalP6.0 at the N-terminus (probability = 0.981). In contrast to CzNIS1a, the CzNIS1b gene was not significantly differentially expressed during infection although its expression pattern exhibited similarities to genes in cluster 5, with expression peaking in the mid stage of infection (**Figure 5C**). CzNIS1b is approximately twice the size of previously studied NIS1 effectors (Yoshino et al., 2012; Chang et al., 2015; Chen et al., 2021; Nie et al., 2022; Di et al., 2024) and contains two putative domains. Alphafold3 modelling of NIS1b predicted a tertiary structure (**Figure 6**) with a beta pleated sheet domain at the N-terminus and alpha helix domain at the C-terminus separated by a serine/threonine-rich linker region. Structural comparison with CoNIS1 identified the N-terminal domain of NIS1b to be structurally similar to CoNIS1 (TM-score. = 0.83). In contrast, the C-terminal domain encoding four consecutive alpha helices does not share structural or sequence homology with proteins of known function, and based on BLASTp analyses appears to be restricted to species in the Mycosphaerellaceae (**Supplementary Figure S5**; **Supplementary Table S5**).

The homolog of the Ecp2 effector in *C. zeina* (BST61_g11601, designated CzEcp2), is encoded by a 569 bp gene containing 2 exons that encodes a 167 amino acid protein with a signal peptide (21 amino acids) predicted by SignalP6.0 at the N-terminus (probability = 0.999) and is predicted as an apoplastic effector by EffectorP 3.0-fungi. The mature sequence of CzEcp2 contains an Hce2 domain (Pfam: PF14856), likely putting it functionally in line with Ecp2 effectors from fungi like *Dothistroma septosporum* and *Fulvia fulva* (Mesarich et al., 2023). To this end, the structures of CzEcp2 and DsEcp2 were predicted using Alphafold3 and compared to one another (**Figure 6**). Each structure was predicted to contain a combination of beta pleated sheets and alpha helices characteristic of the Hce2 domain. Accordingly, structural comparison confirmed that both structures were very similar (TM = 0.84), suggesting that they may serve similar functions *in planta*.

### *C. zeina* candidate secreted effector proteins expressed during the switch to necrotrophy induce cell death in *Nicotiana* spp

**Figure 7:**
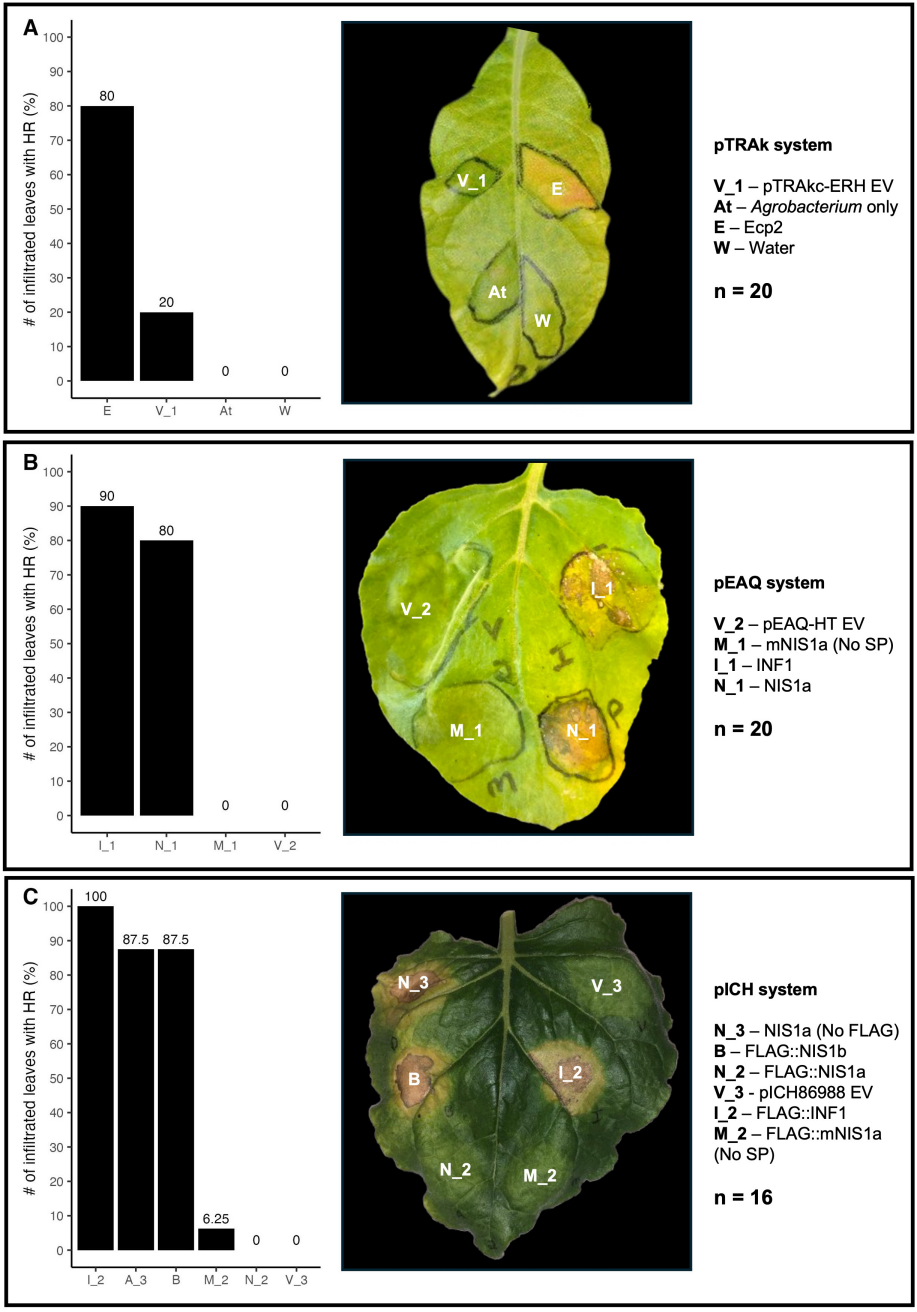
Transient expression of *Cercospora zeina* candidate secreted effector proteins in tobacco species. **(A)** pTRAkc-ERH-mediated transient expression of untagged *C*. *zeina* extracellular protein 2 (Ecp2, E) in *Nicotiana tabacum*. The pTRAkc-ERH empty vector (pTRAkc-ERH EV, V_1), ddH_2_O (Water, W) and *Agrobacterium tumefaciens* without a binary vector (*Agrobacterium*, At) were used as controls. n = 20 leaves were used across 6 plants. **(B)** pEAQ-HT-mediated transient expression of untagged *C*. *zeina* necrosis-inducing secreted protein 1a (NIS1a, N_1) and *C*. *zeina* necrosis-inducing secreted protein 1a mutant with no signal peptide (mNIS1a, M_1) in *Nicotiana benthamiana*. *Phytophthora infestans* INF1 (INF1, I_1) and the pEAQ-HT empty vector (pEAQ-HT EV, V_2) were used as the positive and negative controls, respectively. n = 20 leaves were used across 10 plants. **(C)** pICH86988-mediated transient expression of 3xFLAG tagged (N-terminus) *C*. *zeina* necrosis-inducing secreted protein 1a (FLAG::NIS1a, N_2), *C*. *zeina* necrosis-inducing secreted protein 1a mutant with no signal peptide (FLAG::mNIS1a, M_2), *C*. *zeina* necrosis-inducing secreted protein 1b (3xFLAG::NIS1b, B) and Untagged *C*. *zeina* necrosis-inducing secreted protein 1a (NIS1a (No FLAG), N_3) in *N. benthamiana*. *Phytophthora infestans* INF1 (FLAG::INF1, I_2) and pICH86988 empty vector (EV, V___3) were used as the positive and negative controls, respectively. n = 16 leaves across 8 plants.

Transient expression of CzEcp2 using the pTRAkc-ERH vector system resulted in the induction of cell death in *N. tabacum* (**Figure 7A**). When observed under UV light at 3 dpi, areas transiently expressing CzEcp2 fluoresced with a blueish-white colour, indicating the production of defence-related polyphenolic compounds in response to CzEcp2 (**Supplementary Figure S6**). This phenotype preceded the development of chlorosis which ultimately progressed into cell death at 12 dpi (**Supplementary Figure S6**). Negative controls using distilled water and *Agrobacterium* GV3101 cells (without a binary vector) did not trigger plant immunity, however the negative empty vector control (pTRAKc-ERH) was observed to induce slight chlorosis in a limited number of plants. Western blotting was not conducted for CzEcp2 due to the absence of an epitope tag in the construct.

The transient expression of CzNIS1a using the pEAQ vector system in the leaves of *N. benthamiana* caused cell death similar to the INF1 positive control. However, cell death in areas transiently expressing CzNIS1a occurred between 12 and 14 dpi whereas INF1-induced cell death was apparent between 5 and 7 dpi (**Figure 7B**). The negative empty vector (pEAQ-HT) control did not induce plant immunity. Removal of the signal peptide from CzNIS1a abolished this phenotype (**Figure 7B**), complimenting evidence from previous studies that cell death is mediated by apoplastic recognition of the effector by an unknown receptor (Yoshino et al., 2012; Irieda et al., 2019).

**Figure 8:**
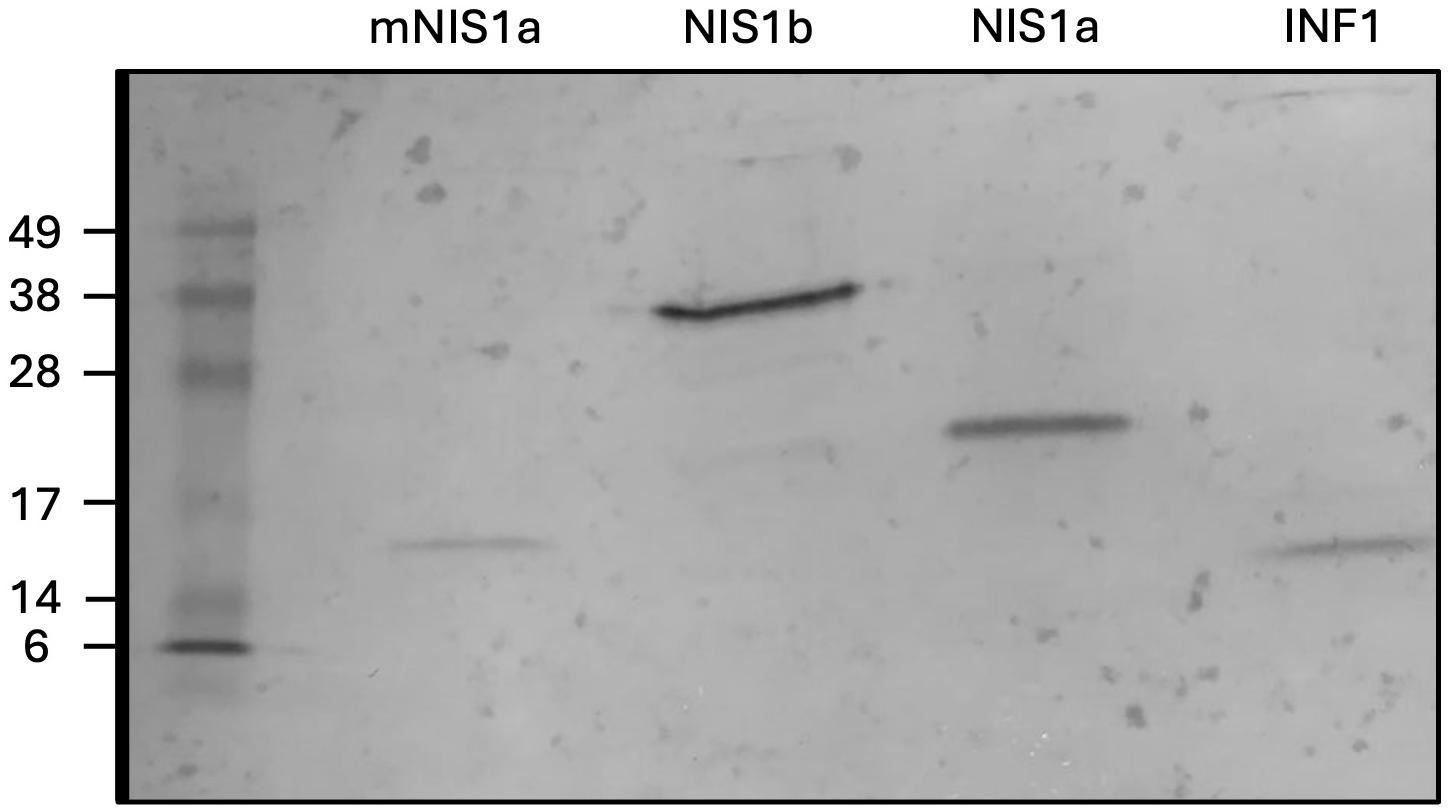
Western blot of 3xFLAG-tagged *Cercospora zeina* candidate secreted effector proteins. The *C. zeina* necrosis inducing secreted protein 1a (NIS1a), necrosis inducing secreted protein 1a mutant without a signal peptide (mNIS1a), necrosis inducing secreted protein 1b (NIS1b) and *Phytophthora infestans* INF1 effectors (INF1) were transiently expressed in *Nicotiana benthamiana* leaves and harvested at 5 days post infiltration. Proteins were extracted and separated using polyacrylamide gel electrophoresis followed by western blotting using anti-3xFLAG antibodies and subsequent detection of chemiluminescence following exposure to 3,3′,5,5′-Tetramethylbenzidine.

**Figure 9:**
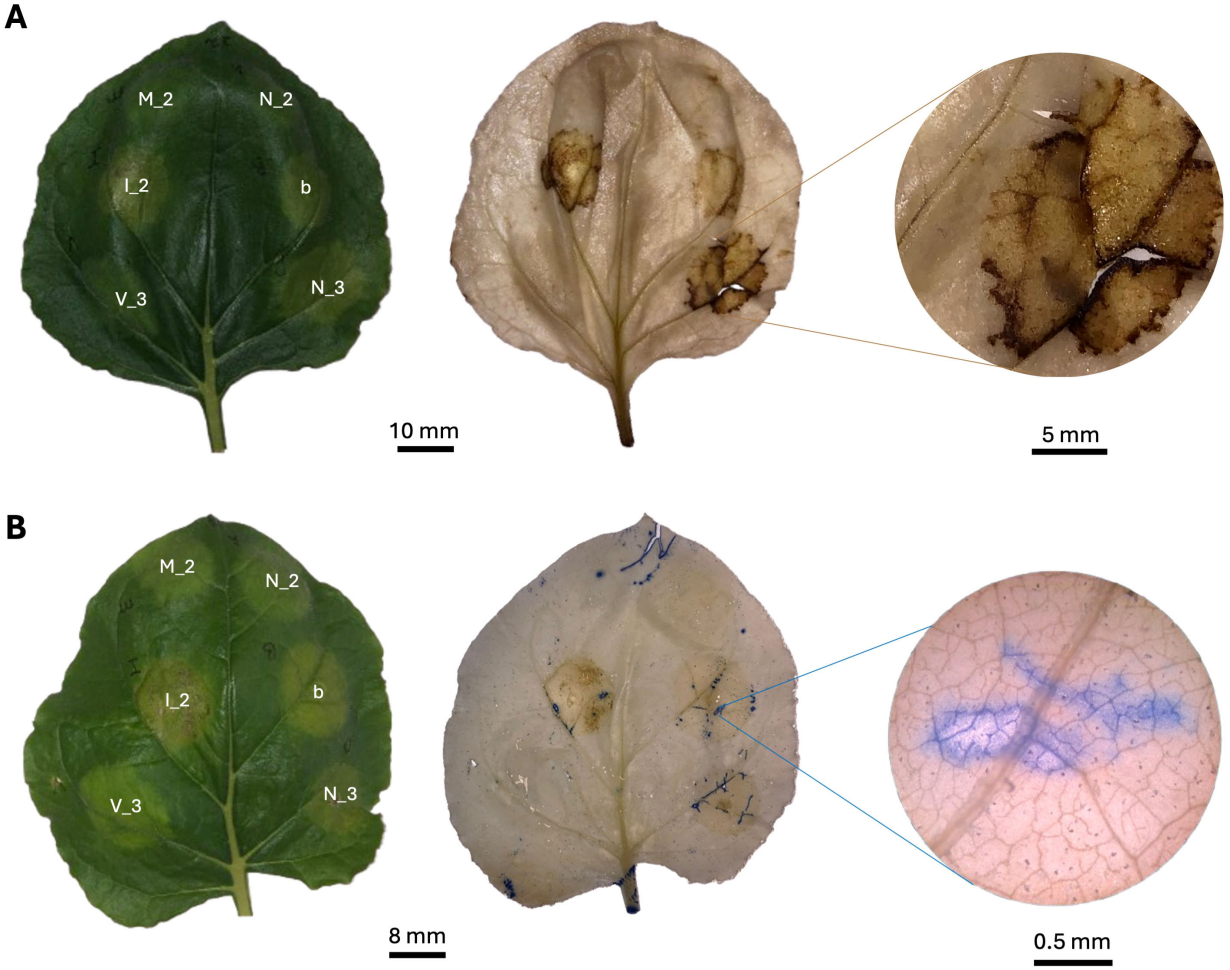
Biochemical assays detecting the presence of reactive oxygen species and callose deposition in *Nicotiana benthamiana* leaves transiently expressing *Cercospora zeina* candidate secreted effector proteins (expressed using the pICH vector system) harvested 7 days post infiltration (dpi), and photographed under white light. **(A)** 3, 3’-diaminobenzidine (DAB) treated leaves showing the formation of a brown precipitate in areas transiently expressing pICH-PR1a::NIS1a, pICH- PR1a::FLAG::NIS1b and pICH- PR1a::FLAG::INF1 indicating the production of ROS in these areas. **(B)** Aniline blue stained leaves showing callose deposition in areas transiently expressing pICH-PR1a::NIS1a (N_3), pICH- PR1a::FLAG::NIS1b (b) and pICH- PR1a::FLAG::INF1 (I_2). Areas infiltrated with pICH-PR1a::FLAG::NIS1a (N_2), pICH86988 EV (V_3) and pICH- FLAG::mNIS1a (No SP) (M_2) did not induce reactive oxygen species production or callose deposition. The DAB-uptake method was carried out as described in Thordal-Christensen et al. 1997.

To confirm production and stability of these proteins *in planta*, a 3xFLAG tag was added to the N-terminus of CzNIS1a and CzNIS1a (No SP) and expressed *in planta* using the pICH86988 vector system. Stability was assessed via Western blotting, followed by recording cell death data. Western blotting of CzNISa and CzNIS1a (No SP) detected bands of approximately 24 kDa and 16 kDa respectively, indicating protein production and stability *in planta* (**Figure 8**). The size of the protein based on the amino acid sequence alone was predicted to be 16.8 kDa. The size difference between CzNIS1a and CzNIS1a (No SP) likely represents post-translational differences between the proteins, a finding similarly observed for CoNIS1 (Yoshino et al., 2012). Surprisingly, addition of the 3xFLAG tag to CzNIS1a appeared to interfere with its phenotype and did not cause cell death despite (**Figure 7C**) the 3xFLAG::CzNIS1a and 3xFLAG::CzNIS1a (No SP) proteins being produced *in planta* (**Figure 8**). In contrast, the phenotype of the INF1 positive control (pICH-INF1) (**Figure 7C**) was not affected by the addition of the 3xFLAG tag and was similarly detected by Western blotting (**Figure 8**). Removal of the 3xFLAG tag from 3xFLAG::CzNIS1a followed by transient expression of the untagged CzNIS1a recovered the original phenotype (**Figure 7C**). Subsequent staining experiments using the untagged variant (in the pICH vector system) showed that CzNIS1a could induce ROS generation and callose deposition within infiltrated areas prior to the development of cell death (**Figure 9**).

The CzNIS1b CSEP was similarly investigated in leaves of *N. benthamiana* and resulted in the development of cell death within infiltrated areas (**Figure 7C**). Unlike CzNIS1a and the INF1 positive control, areas transiently expressing CzNIS1b only developed a cell death phenotype after 18 dpi. The CzNIS1b phenotype was seemingly unaffected by the addition of the 3xFLAG tag as ROS generation and callose deposition were observed (**Figure 9**) in leaves harvested at 7 dpi, respectively. Western blotting of 3xFLAG::NIS1b detected an approximately 36 kDa band (**Figure 8**) that was larger than the size expected from the amino acid sequence alone (32 kDa), likely indicative of post-translational modifications.

## Discussion

In this work, gene expression was examined across maize samples harvested from the early, mid and late stages of GLS infection to determine if the lifestyle of *C. zeina* represents that of a latent necrotroph. Analysis of differentially expressed genes revealed two waves of pathogen gene expression across infection. The first wave occurred during the early stage of disease and was associated with low levels of disease symptoms, reduced biomass accumulation and low levels of metabolic activity, with glycosyltransferase, CSEP and cell cycle genes representing the majority of gene expression during this wave. Symptom development occurred in the mid and late stages of disease and corresponded to increased levels of pathogen biomass accumulation. Both stages also showed similar gene expression trends and thus together constituted the second wave of gene expression in which detoxification, glycosyl hydrolase, and pectinase genes were highly expressed. Most CSEP genes were expressed during this wave, and three CSEPs with homology to effectors known to perturb host immunity were identified among genes expressed upon the switch to necrotrophy. CzEcp2, CzNIS1a and CzNIS1b were subsequently transiently expressed in model plant systems and shown to trigger non-host resistance, suggesting they target conserved structures or processes to facilitate pathogen colonization and growth during the switch to necrotrophy.

### The switch to necrotrophy coincides with a transition to rapid pathogen growth

The number of RNA-seq reads mapping to a pathogen genome has been correlated with pathogen biomass in several ascomycete phytopathogens (Blanco-Ulate et al., 2014; Lanver et al., 2018; Human et al., 2020) and was thus used to assess the biomass accumulation of *C. zeina* during infection. In line with data from earlier GLS studies, *C. zeina* biomass was found to increase during infection and was strongly correlated with symptom development (Korsman et al., 2012; Swart et al., 2017). Upon symptom development in the mid and late stages, markedly higher levels of pathogen biomass were observed, with a more than 40-fold increase in pathogen biomass recorded between the early and late stages, suggesting that growth restraints observed in the early stage are relaxed in the late stage. Results from the transcriptome analysis complemented this observation and showed that two contrasting waves of gene expression take place before and after lesion development occurs, highlighting a transcriptional switch that takes place between the early and late stages of infection. Altogether, these results show that *C. zeina* accumulates relatively little biomass during the latent period and that the majority of fungal growth takes place following symptom development, a pattern similarly observed in the latent necrotroph, *Z. tritici* (Sánchez-Vallet et al., 2015).

### The first wave of gene expression suggests *C. zeina* is quiescent during the latent period

Upon landing on a leaf, *C. zeina* conidia germinate and quickly enter the stomata (Marais et al., 2024), after which the pathogen enters a seemingly quiescent period. Gene expression during this wave was heavily overrepresented by gene ontologies involved in cell cycle regulation, chromosome organisation and DNA replication along with various growth-related processes involving glycosyltransferase (GT) families. This shows that there is an emphasis on genes involved in cellular growth and development (Hasby et al., 2021) and suggests that the pathogen adopts a restricted growth strategy that results in substantially lower biomass accumulation when compared to the second wave of gene expression.

A total of six hydrophobins were identified from the genome of *C. zeina*, with four of them showing peak expression in the early stage of disease. Hydrophobins are known to interact with fungal cell walls to modulate the way in which the pathogen interacts with host tissues (Whiteford and Spanu, 2002; Lopatukhin et al., 2024; Rojas-Osnaya et al., 2024). Previous work has postulated that hydrophobins from pathogens like *Cladosporium fulvum* and *Z. tritici* serve as effectors that play a role in hiding the pathogen from plant immunity (Whiteford and Spanu, 2002; Sánchez-Vallet et al., 2015), a phenomenon observed in medically relevant pathogens like *Aspergillus fumigatus* (Hernández-Chávez et al., 2017). In light of these observations, it is plausible to suggest that the early expression of hydrophobin genes in *C. zeina* facilitates the establishment of fungal hypha and conceals the fungus, thereby avoiding premature activation of host defences and allowing the pathogen to develop undetected.

Chitin binding effector genes have been shown to play a role in hiding pathogens and protecting them from plant defences (Stergiopoulos et al., 2010; Marshall et al., 2011). Although *C. zeina* homologs of the Ecp6 and Avr4 effectors were not differentially expressed in the experiment, their constitutive expression across all stages of infection likely highlights the important role they play in protecting the pathogen from host defences. Although chitin-binding genes and hydrophobins only represent a subset of the CSEP genes expressed during the early stage, their expression patterns and putative functions highlight a trend which suggests the pathogen is trying to evade host defences in this stage.

Altogether, the relatively low amount of biomass combined with the lack of symptom development, expression of hydrophobins, chitin binding proteins and growth-related GTs as well as limited expression of CWDEs suggests that *C. zeina* prioritises stealth over growth during the latent period. Furthermore, the unusually long latent period coupled with the lack of haustoria-like structures (Beckman and Payne, 1982) typically associated with classic hemibiotrophic pathogens such *Magnaporthe oryzae*, suggests that *C. zeina* adopts more of an endophytic behaviour (Kaczmarek et al., 2017; Collinge et al., 2022) rather than that of a traditional hemibiotroph.

### The second wave of gene expression is characteristic of necrotrophy

Plant cell walls are predominantly composed of cellulose (Kozlova et al., 2020; Delmer et al., 2024). In cereal crops like maize, cellulose is typically supported by hemi-cellulosic molecules like xylans, xyloglucans and other mixed linkage glucans (Kozlova et al., 2020; Delmer et al., 2024). The upregulation of pathogen GH genes involved in cell wall degradation during the mid and late stages of disease suggests *C. zeina* uses cell wall degrading enzymes (CWDEs) like cellulases and xylanases to degrade cell walls in infected tissues to release energy-rich compounds. The expression of these genes is typical of the necrotrophic stage (Bradley et al., 2022) and previous studies have shown that the upregulation of xylanases and amylases from various GH families facilitates the release of high energy carbon compounds that support pathogen growth and proliferation (Paolinelli-Alfonso et al., 2016; Moreno-Sánchez et al., 2021).

Despite their reduced abundance, pectins have also been shown to play important structural and defensive roles in the cell walls of monocots (Kozlova et al., 2020; Huang et al., 2023). The differential expression of twelve genes involved in pectin degradation across infection shows that *C. zeina* also uses CWDEs to target pectin degradation during infection. Previous work in *Magnaporthe oryzae* identified a pectate lyase called MoPL1 that was highly expressed in the mid to late stages of disease development and was shown to facilitate the movement of infective hyphae through adjacent cells (Wegner et al., 2022). This observation was similarly noted in another monocot pathogen, *Fusarium sacchari*, in which a pectate lyase mutant showed impaired penetrative capabilities and reduced virulence compared to the wild-type, suggesting that pectinase activity supports hyphal penetration of cell walls in sugarcane (Wang et al., 2023).

Given these observations, it is likely that *C. zeina* uses its suite of pectinases to support the invasion of host cells during its necrotrophic stage. The highly expressed BST61_g5290, which shows a high degree of structural similarity to the pectinase VdPEL1, likely supports the movement of infective hyphae by weakening plant cell walls during the mid and late stages of infection. Expression of a different set pectinases in the early stage of infection likely allows the pathogen to colonise tissues in a more subtle manner that does not trigger immunity (Sánchez-Vallet et al., 2015); a feature not required following symptom development, hence their downregulation in the second wave of gene expression. This hypothesis may also explain why some GH genes are downregulated following the first wave of gene expression.

The degradation of plant tissues releases damage-associated molecular patterns (DAMPs) which trigger plant defences (Hou et al., 2019; Ma et al., 2024). As a result, plants produce harmful molecules like reactive oxygen species to restrict pathogen development and prevent further colonisation (Hou et al., 2019; Farvardin et al., 2024). To protect themselves, fungi like *C. zeina* are thought to produce protective proteins and enzymes like glutathione-s-transferases (GSTs) to detoxify reactive oxygen species and ameliorate host defences (Calmes et al., 2015; Wangsanut and Pongpom, 2022). Detoxification genes and GST pathways were significantly upregulated in the pathogen and showed high levels of expression following symptom development in the mid and late stages of disease. This pattern also mirrored the expression of the degradative GH enzymes and thus indicates a link between degradation and detoxification pathways during infection. Mid to late expression of genes with these functions is characteristic of necrotrophs (Gullner et al., 2018), and highlights *C. zeina’s* need for detoxifying gene products as it breaks down maize tissues (Calmes et al., 2015) to support its growth and development.

The conclusion of the fungal lifecycle results in the production of conidiophores from which conidia are released (Jung et al., 2014). The hydrophobins BST61_g1984 and BST61_g6910 show very high levels of gene expression in the second wave which coincides with mature lesion development and sporulation. BST61_g6910 shows protein structure similarity to Cryparin from *Cryphonectria parasitica*, whereas BST61_g1984 shows protein structure similarity to MPG1 from *Magnaporthe oryzae*. Studies have shown that these hydrophobins typically have functions that are critical for conidiophore formation, sporulation and spore germination (Whiteford and Spanu, 2002; Kim et al., 2005; Pham et al., 2016; Quarantin et al., 2019). Consistent with this, the *C. zeina* hydrophobins BST61_g1984 and BST61_g6910 are likely to have putative functions in the previously mentioned processes and that these processes occur as a result of the switch to necrotrophy.

### Fungal effectors are expressed to facilitate the switch to necrotrophy

In addition to detoxifying enzymes, fungi also produce effectors to facilitate infection by modulating the host-pathogen interaction (Stergiopoulos and Wit, 2009; Seong and Krasileva, 2023). Throughout infection, many *C. zeina* CSEPs were differentially expressed but more than half showed expression peaks in the mid stage of disease, exemplified by cluster 5 which contained 23 CSEPs with very similar expression patterns. Since the average expression pattern of cluster 5 coincided with the symptom development in the mid stage, CSEP genes in the cluster were hypothesized to modulate plant cell function to support the change in pathogen lifestyle. Examination of this hypothesis revealed the presence of CzNIS1a, CzNIS1b and CzEcp2, all of which are homologous to effectors known to activate immunity in non-host species as a result of their activity (Stergiopoulos et al., 2010; Irieda et al., 2019).

Upon transient expression in *N. tabacum*, CzEcp2 invoked a strong defence response resulting in the development of severe chlorosis and necrosis characteristic of a programmed cell death response. Activation of defence responses were additionally monitored under UV light. Infiltrated areas expressing CzEcp2 initially displayed dark red fluorescence, characteristic of chlorophyll autofluorescence (Donaldson, 2020). With time, the dark red fluorescence evolved into blue-white colour, indicating the accumulation of phenolic compounds typically associated with plant defence and/or cell death (Kettles et al., 2017). The phenotype of CzEcp2 in *N. tabacum* is similar to the phenotypes documented for Ecp2 effector homologs from *Cladosporium fulvum, Dothistroma septosporum*, and *Mycosphaerella fijiensis* (De Kock et al., 2004; Stergiopoulos et al., 2010; Guo et al., 2020). Based on the structural similarity between CzEcp2 and DsEcp2, it is likely that both effectors function in similar ways, and as a result of their activity, trigger plant immunity by activating the Cf-Ecp2-like immune receptor in *N. tabacum* (Stergiopoulos et al., 2010; Guo et al., 2020).

Transient expression of CzNIS1a and CzNIS1b demonstrated that both effectors were able to activate plant immunity by inducing ROS production, callose deposition and eventually cell death when expressed in *N. benthamiana*. Removal of the signal peptide from CzNIS1a abolished the cell death phenotype and suggests plant defences are activated by a receptor capable of recognizing CzNIS1a in the apoplastic space but not in the cytoplasm; an observation similarly noted during the investigation of CoNIS1 (Yoshino et al., 2012). Apoplastic recognition of NIS1 effectors is thought to be mediated by a functional domain present in the last 30 amino acids at the C-terminal end of the NIS1 protein (Irieda et al., 2019). In the *Magnaporthe oryzae* and *Valsa mali* pathosystems, intrinsic truncation of the MoNIS1 and VmNIS2 effectors at the C-terminus allows both effectors to escape recognition when delivered to the apoplast (Irieda et al., 2019; Nie et al., 2022). Despite the extended C-terminus of CzNIS1b and the presence of a unique protein domain in this region, it is possible that CzNIS1b activates non-host plant defences in a similar way to CzNIS1a and that this activation may aid the necrotrophic lifestyle of the pathogen.

Although intrinsic differences in terms of size and expression level exist between CzNIS1a and CzNIS1b, the structural conservation of the N-terminus suggests they may have related functions in disrupting cellular function. When undetected, NIS1 effectors have been shown to modulate several defence pathways *in planta* by targeting factors involved in signal transduction and ROS production (Irieda et al., 2019). This is demonstrated by CoNIS1’s ability to suppress INF1 induced cell death in *N. benthamiana* by targeting BAK1 and BIK1, two components of PTI which are vital for initiating plant defences upon pathogen detection (Franco-Orozco et al., 2017; Irieda et al., 2019). NIS1 interacts with and inhibits the kinase activities of BAK1 and BIK1 thereby preventing their autophosphorylation (Irieda et al., 2019; Nie et al., 2022). This prevents the activation of downstream signalling pathways (Chinchilla et al., 2009; Shang et al., 2021) and also prevents the activation of respiratory oxidase homolog D (RBOHD), which suppresses ROS bursts and downstream signalling processes associated with ROS production (Irieda et al., 2019; Chen et al., 2021; Nie et al., 2022).

Although cell death induction is likely not the function of the CzNIS1a, CzNIS1b and CzEcp2 effectors in *Nicotiana* spp, their ability to trigger plant immunity suggests that they target conserved cellular structures or processes for which receptors have evolved in non-hosts to detect their presence (Chen et al., 2021). Given the phylogenetic distance between *Zea mays* and *Nicotiana* spp., it is likely that these receptors are absent in maize, allowing the effectors to execute their functions and support *C. zeina* during its transition to necrotrophy.

## Conclusion

The fungus *C. zeina* has a long latent period in which no symptoms of gray leaf spot disease of maize are detectable. Gene expression analyses showed that the pathogen is relatively inactive during this period and that the majority of genes involved in growth and metabolism were only expressed upon the switch to necrotrophy when symptoms developed. This pattern was similarly observed amongst the CSEP genes, with most CSEP genes being expressed upon or after the switch to necrotrophy. Altogether, the information in this study shows that the emphasis of the pathogen’s lifestyle is on necrotrophy, and like *Z. tritici* the pathogen is better described as a latent necrotroph.

## Data Availability

The data presented in the study are deposited in the NCBI gene expression omnibus (GEO) and GenBank repositories, accession numbers GSE305815 and GCA_002844615.3, respectively.
