# Supplementary material for: A latent invader: transcriptomics reveals Cercospora zeina’s stealth infection strategy of maize and immune-activating effectors
Source: Front Plant Sci. 2025 Nov 7;16:1703682. doi: 10.3389/fpls.2025.1703682 (PMC12676973; doi:10.3389/fpls.2025.1703682)
Supplement: Supplementary file 1 [file DataSheet1.pdf]

Supplementary Figures and Tables

A latent invader: Transcriptomics reveals *Cercospora zeina*'s stealth infection strategy of maize and immune-activating effectors

Frontiers in Plant Science, Plant Pathogen Interactions, Manuscript #: fpls.2025.1703682

Trystan Nadasen[1], Carla Buitendag[1], Rodé Visser[1], Tanya Welgemoed[1], Ingo Hein[2,,3*], Dave Berger[1*]

[1]Department of Plant and Soil Sciences, Forestry and Agricultural Biotechnology Institute (FABI), University of Pretoria, Pretoria, South Africa

[2]Potato Disease Resistance group, James Hutton Institute, Dundee, Scotland

[3]School of Life Sciences, University of Dundee, Dundee, United Kingdom

*Corresponding authors

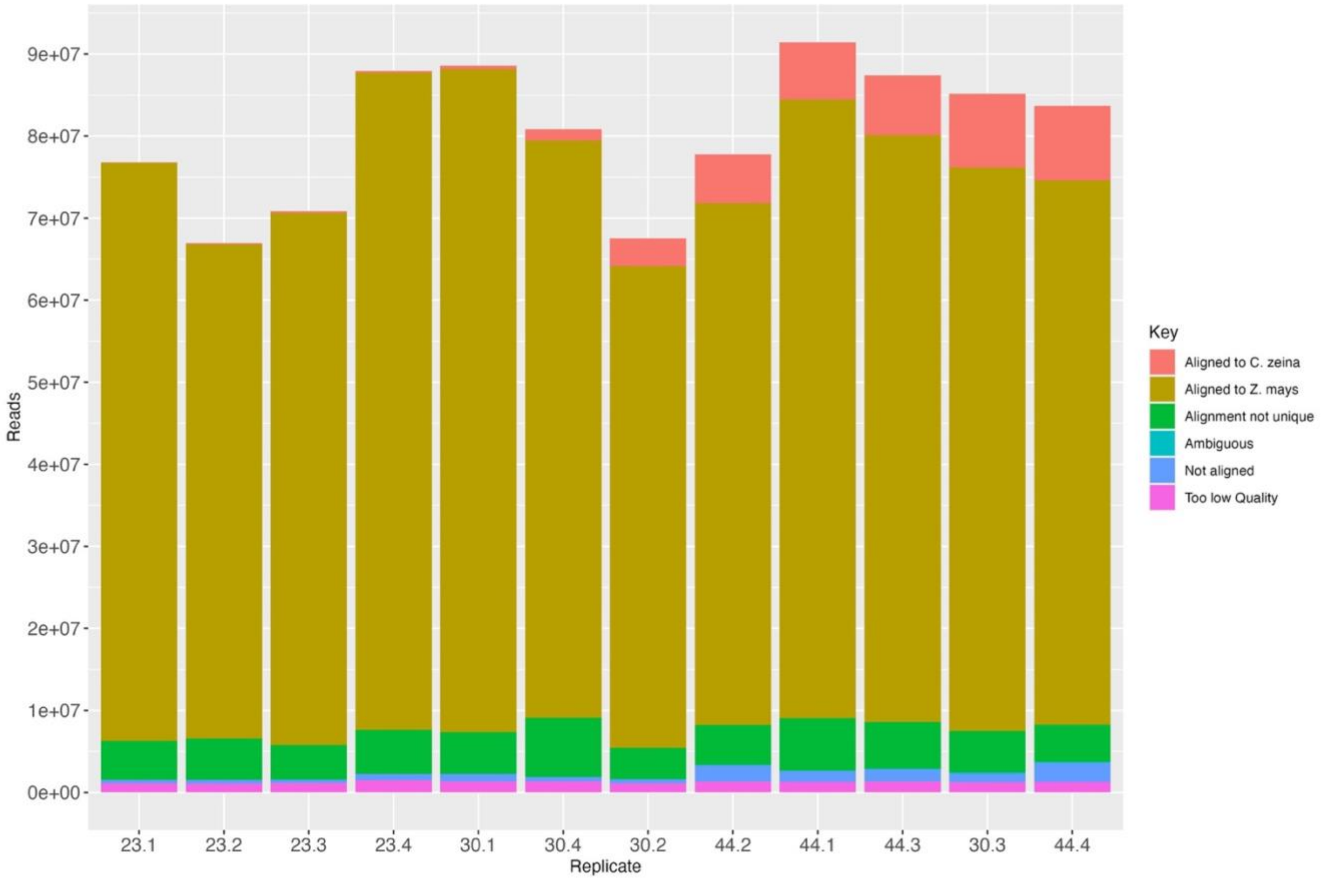

Figure S1. Summary of the RNA sequencing reads processed by HTseq-count following the mapping of reads to the concatenated *Cercospora zeina – Zea mays* genome.

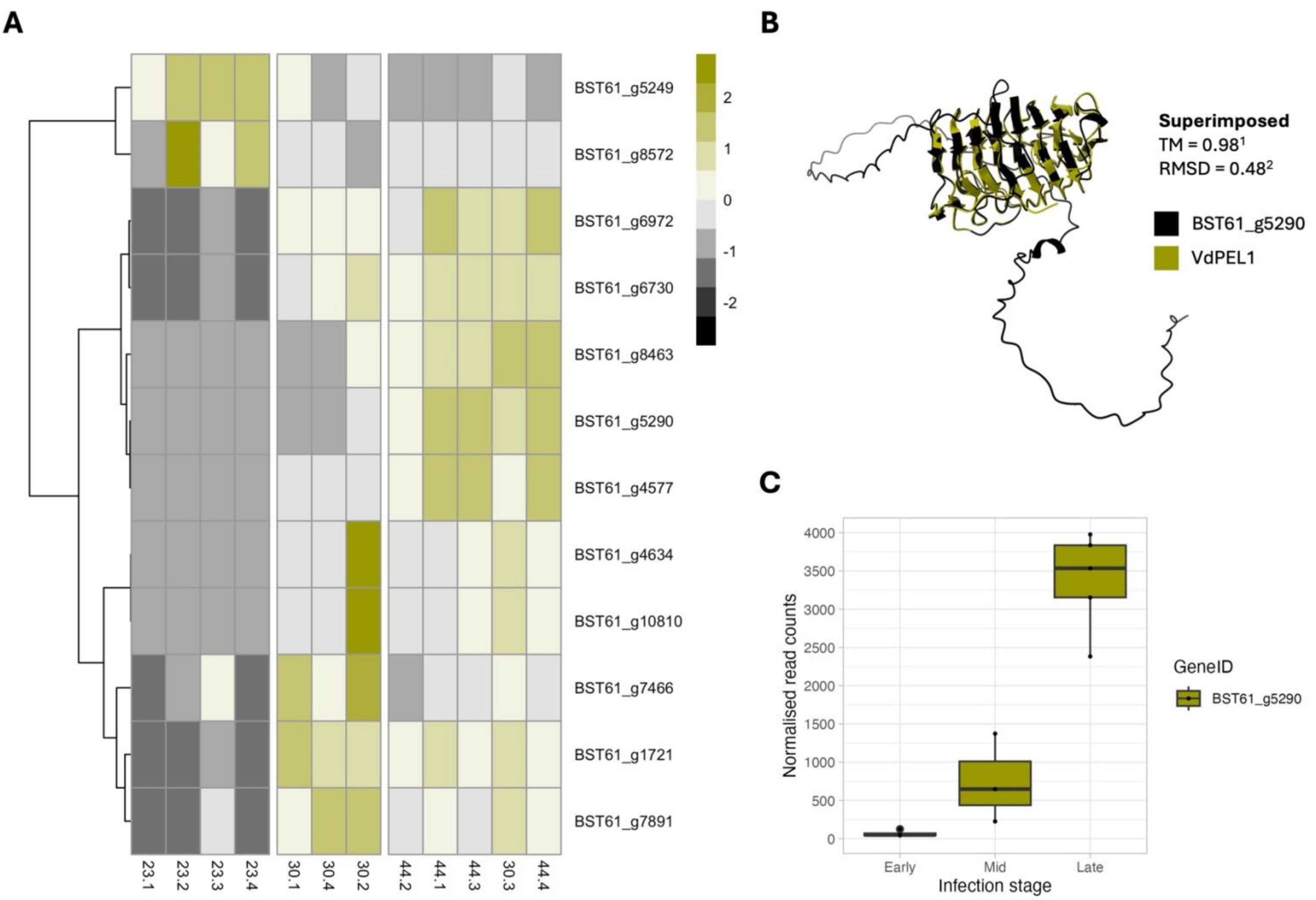

Figure S2. *Cercospora zeina* genes predicted to function in pectin degradation. (**A**) Heatmap of differentially expressed *C. zeina* genes involved in pectin degradation showing their expression across the early (23 dpi), mid (30 dpi) and late stages (44 dpi) of grey leaf spot disease on maize. (**B)** Alphafold3 predictions of the BST61_g5290 and *Verticillium dahliae* PEL1 proteins superimposed on one another using ChimeraX (version1.9). (**C**) Expression of the BST61_g5290 gene in the three stages of disease. Genes that are not significantly differentially expressed (adjusted p-value < 0.05 and log2FC > 1 or log2FC < -1) in the Late-Early, Mid-Early and/or Late-Mid contrasts are represented by the same letter. a

[1]TM score relative to the shortest protein, VdPEL1

[2]RMSD score based on pruned atoms pairs

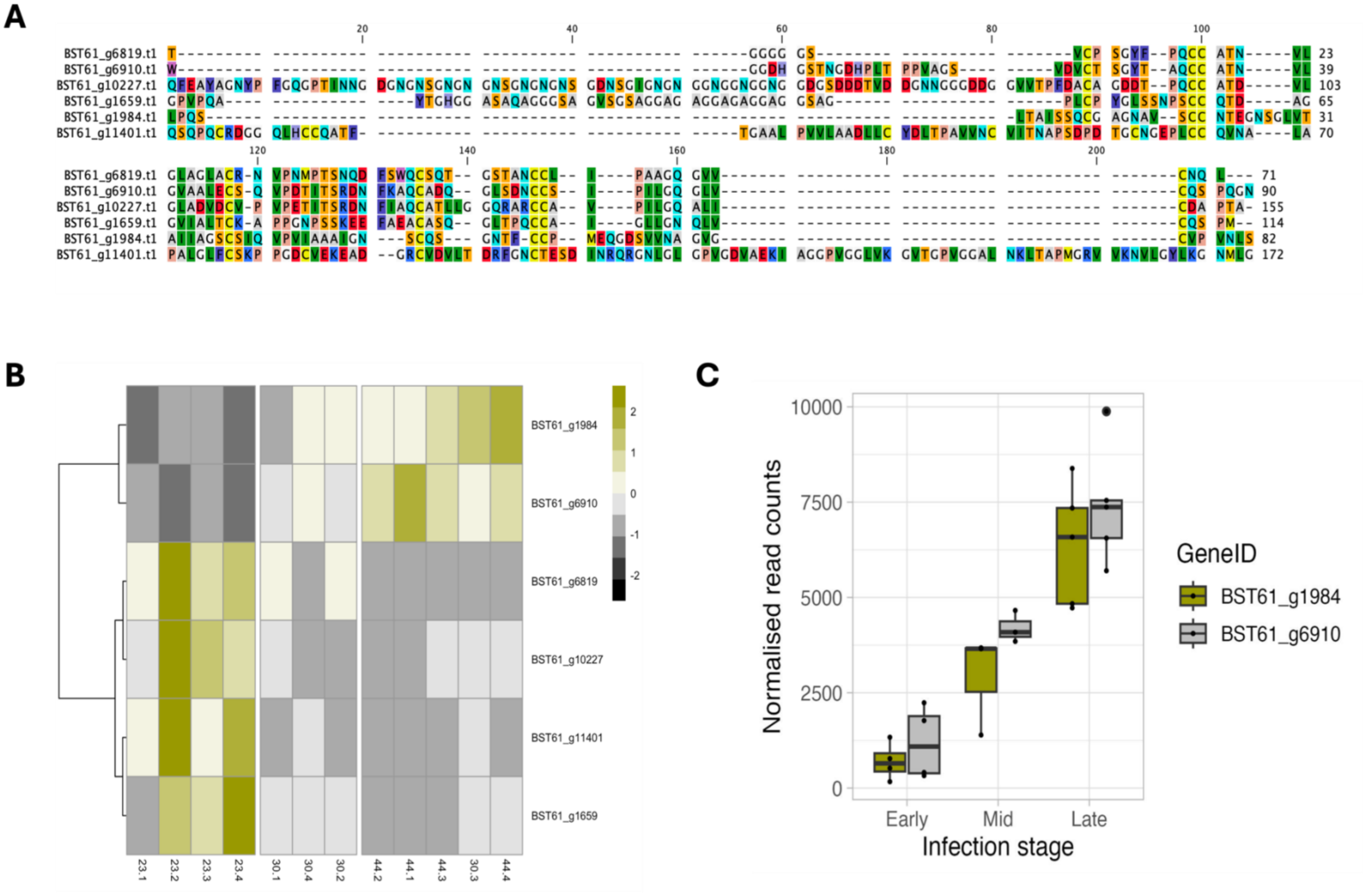

Figure S3. *Cercospora zeina* genes predicted to function as hydrophobins. (**A**) Alignment of mature regions of differentially expressed *C. zeina* hydrophobin genes using CLC main workbench 23 with conserved cysteine residues highlighted in yellow. (**B**) Heatmap of differentially expressed *C. zeina* hydrophobin genes showing their expression across the early (23 dpi), mid (30 dpi) and late stages (44 dpi) of grey leaf spot disease on maize. (**C**) Expression patterns of the BST61_g1984 and BST61_g6910 hydrophobin genes that are highly upregulated during infection.

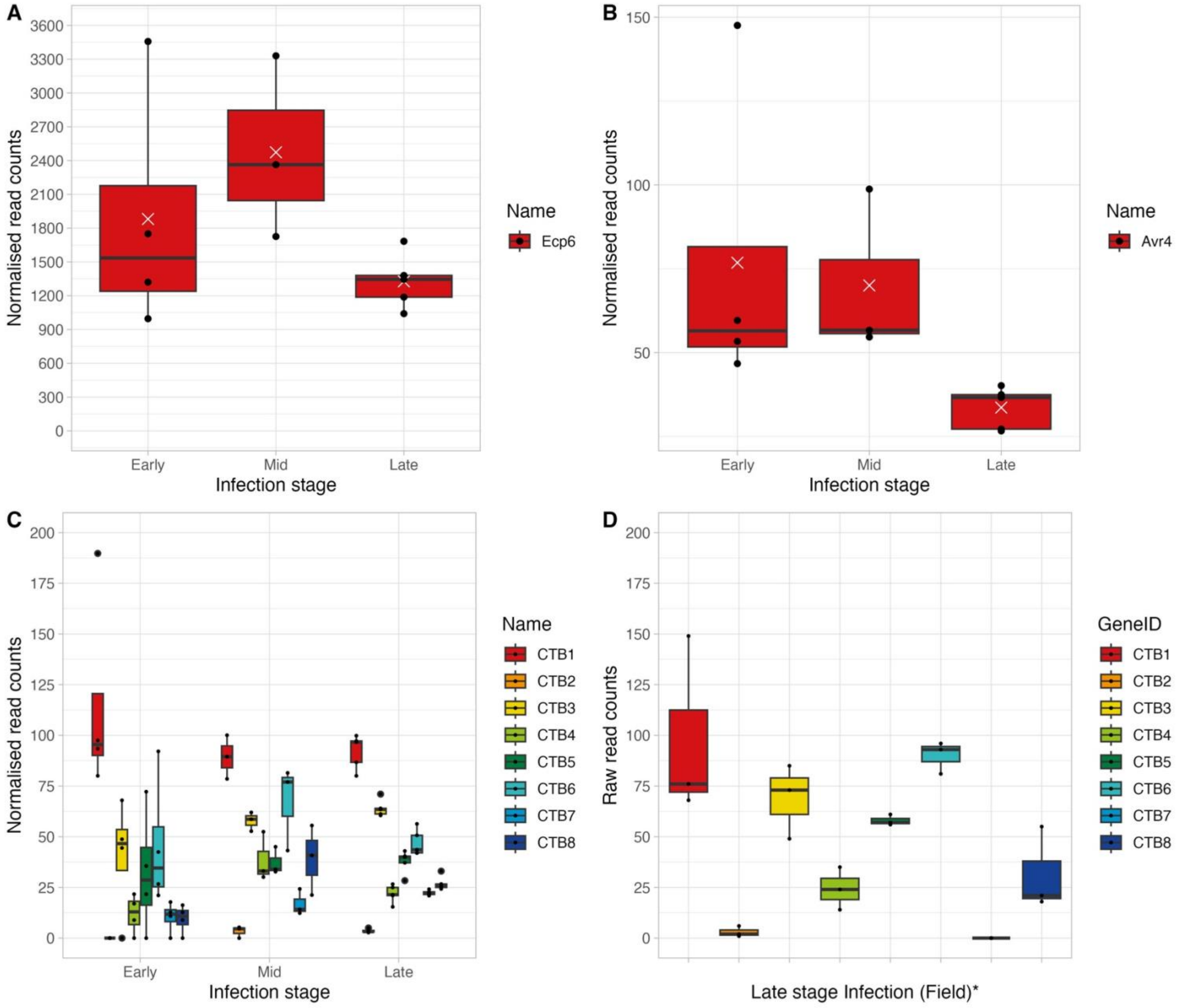

E

	Field	Cornmeal agar	V8 agar	Complete media	YPD media
**Early (23 dpi)**	0.84	0.85	0.85	0.85	0.53
**Mid (30 dpi)**	0.94	0.73	0.75	0.72	0.57
**Late (44 dpi)**	0.88	0.88	0.89	0.87	0.74

Figure S4. Expression patterns of secondary metabolism and chitin-binding genes from *Cercospora zeina* during the infection of maize. (**A**) Expression of the CzEcp6 gene involved in chitin-binding in the early, mid and late stages of GLS disease. (**B**) Expression of the CzAvr4 gene involved in CTB gene regulation and chitin-binding in the early, mid and late stages of GLS disease. (**C**) The expression patterns of the eight genes in the cercosporin toxin biosynthesis (CTB) gene cluster (CTB1-8) in the early, mid and late stages of GLS disease in B73 maize grown in a conviron. (**D**) Data from Swart *et al.* (2017) showing the expression patterns of the eight genes in the CTB gene cluster (CTB1-8) in the late stages of GLS disease in field grown B73 maize. (**E**) Pearson correlation coefficients calculated between the conviron data (early, mid and late groups) and the field/ *in vitro* data. YPD: yeast extract, peptone and dextrose media.

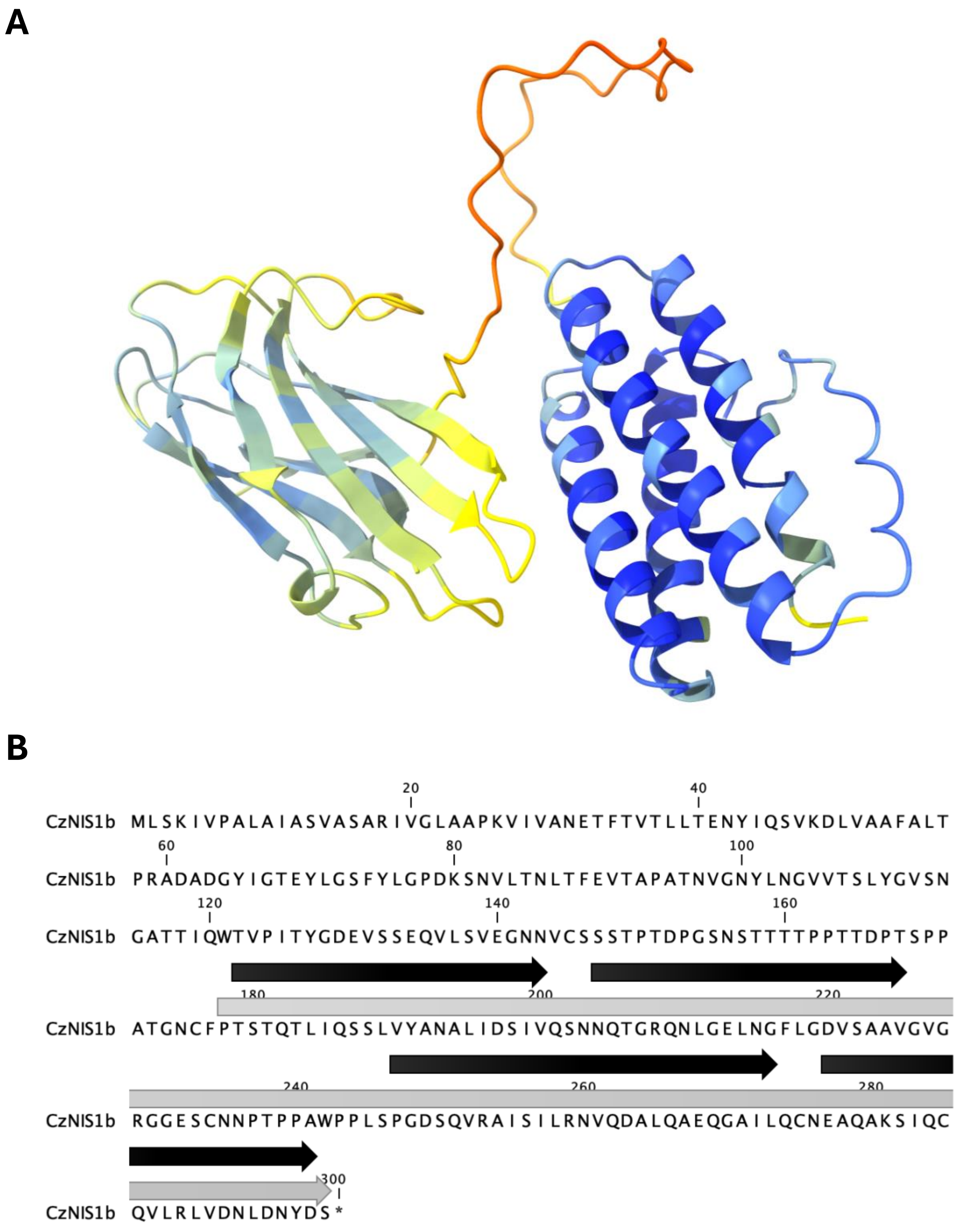

Figure S5. Protein and sequence domains of the *Cercospora zeina* Necrosis-inducing secreted protein 1b (CzNIS1b). (**A**) Alphafold3 prediction of the CzNIS1b protein highlighting the four alpha helices at the C-terminal end of the protein. (**B**). Amino acid sequence of the CzNIS1b protein. The C-terminal domain with unknown function constitutes the last 122 amino acids of the protein and is represented by the grey arrow. The sequence of the four alpha helices that make up the domain are represented by black arrows.

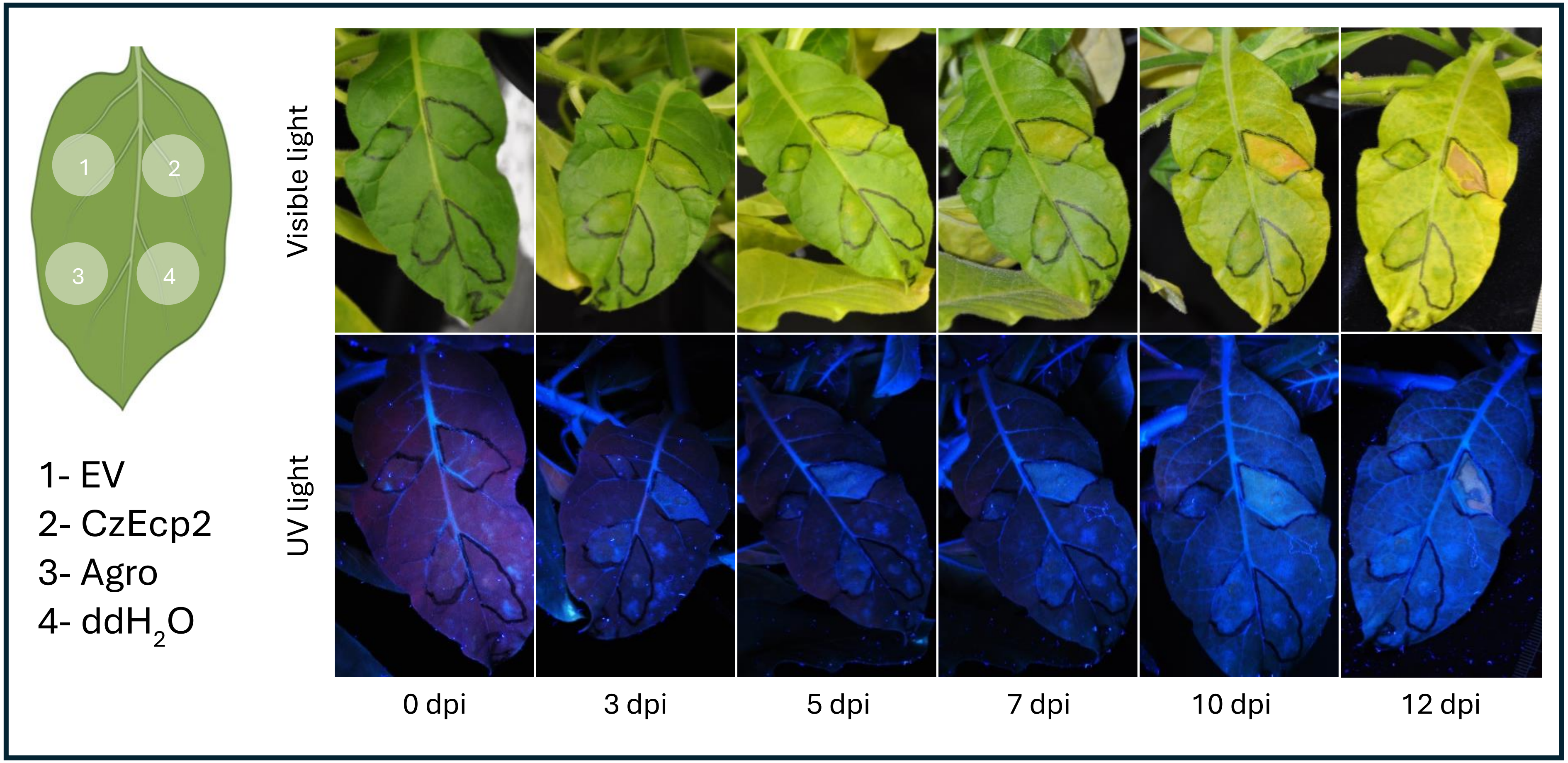

Figure S6. Photographs capturing the immune response of CzEcp2 in *Nicotiana tobacum* leaves from 0 to 12 days post infection (dpi) following transient transformation using *Agrobacterium* GV3101 cells containing pTRAkc-ERH (EV), pTRAk-CzEcp2 (CzEcp2), *Agrobacterium* GV3101 only (Agro), or water control (ddH_2O) respectively. The photographs were taken under normal spectra light and UV light.

Table S1. Primers used to amplify *Cercospora zeina* effector sequences

Primer	**Purpose***	Sequence (5' to 3')	Tm (°C)
pQNIS1-R	OE-PCR	ATATCTCGAGTCAGTTCTTGACATCTGGAAC	58
NIS1-Fpa		CCACTCTTGCCGTGCCCGCATCGTCGGCTTC	58
NIS1-Rpa		TCAGTTCTTGACATCTGGAACCG	60
pQNIS1-F		CGAGACCGGTATGCGCATCGTCGGCTTCTAC	60
PR1a-3xFLAG_F	Golden gate	GGCTACGGTCTCTAATGGGATTTGTTCTCTTTTCACAATTGC	70
NIS1a2-R		GGCTACGGTCTCTTAGAAGCCGACGATGCGCTTGTCGTCGTCGTCCTTG	70
NIS1a3-F		GGCTACGGTCTCTTAGAAGCCGACGATGCGCTTGTCGTCGTCGTCCTTG	70
NIS1a4-R		GGCTACGGTCTCCAAGCTCAGTTCTTGACATCTGGAAC	70
FmNIS1a-F		GGCTACGGTCTCTAATGCAAAATGACTACAAGGACCACGACGG	70
NIS1-F	cDNA	ATGCGTGCCTCTATCCTC	60
NIS1-R		TCAGTTCTTGACATCTGGAACC	60
Ecp2F	cDNA	ACCACACTCCTCCACCAAGA	58
Ecp2R		TCCACCAGCAGCGCATACTC	58
CzEcp2-NcoI F	RE	CAGCCATGGTCCCACAGAGGAAGAA	57
CzEcp2-BamHI R		AGTGGATCCCTAGTTCGATGGGTTGTA	57

*Intended use of primer. OE-PCR: Primers used to prepare sequences for overhang extension PCR; Golden gate – Primers used to prepare sequences for golden gate cloning; cDNA: Primers used to isolate effector sequences from cDNA; RE.- Primers used to add restriction enzyme cut sites to effectors

Table S2. Reads mapping to the *Zea mays* and *Cercospora zeina* genome as well as the percentage area of each leaf sample showing gray leaf spot disease (GLS) symptoms

**Disease stage**	**Early**				**Mid**				**Late**			
Sample[1]	23.1	23.2	23.3	23.4	30.1	30.4	30.2	30.3	44.1	44.2	44.3	44.4
Total reads	76,814,328	66,938,713	70,847,870	87,960,990	88,593,600	80,846,368	67,560,810	85,144,073	91,454,312	77,772,261	87,396,752	83,696,856
% reads mapping to *Z. mays* genome	91.71	90.00	91.56	91.04	91.21	87.04	86.91	80.61	82.44	81.79	81.80	79.25
% reads mapping to *C. zeina* genome	0.06	0.18	0.29	0.29	0.47	1.71	5.07	10.56	7.66	7.66	8.35	10.86
% leaf area showing GLS symptoms	2	3	10	18	11	31	31	73	57	62	61	76

[1]Samples ordered by the percentage of reads mapped to the *C. zeina* genome within the early, mid and late stages of disease.

Table S3. Differentially expressed carbohydrate-active enzymes from the glycoside hydrolase and glycosyltransferase enzyme classes.

Gene	Description[1]	CAZy[2]	PFAMs[3]	Regulation
BST61_g2365.t1	Glycoside hydrolase 1	GH1	Glyco_hydro_1	Upregulated
BST61_g10268.t1	Glycoside hydrolase 10	-	Glyco_hydro_10	Upregulated
BST61_g2276.t1	Glycoside hydrolase 11 (cellulase G)	-	Glyco_hydro_11	Upregulated
BST61_g2007.t1	Glycoside hydrolase 11 (cellulase G)	-	CBM_1, Glyco_hydro_11	Upregulated
BST61_g2529.t1	Glycoside hydrolase 115	-	Glyco_hydro_115	Upregulated
BST61_g3939.t1	Glycoside hydrolase 12 (cellulase H)	GH12	Glyco_hydro_12	Upregulated
BST61_g1609.t1	Glycoside hydrolase 12 (cellulase H)	GH12	Glyco_hydro_12	Upregulated
BST61_g7838.t1	Glycoside hydrolase 13	GT5	Alpha-amylase, Glyco_trans_1_4, Glyco_transf_5, Glycos_transf_1	Upregulated
BST61_g827.t1	Glycoside hydrolase 13	CBM48, GH13	Alpha-amylase, Alpha-amylase_C, CBM_48	Upregulated
BST61_g10672.t1	Glycoside hydrolase 131	-	CBM_1	Upregulated
BST61_g4215.t1	Glycoside hydrolase 17	-	Glyco_hydro_17	Upregulated
BST61_g5357.t1	Glycoside hydrolase 18	-	Glyco_hydro_18, UBA	Upregulated
BST61_g903.t1	Glycoside hydrolase 2	-	Glyco_hydro_2, Glyco_hydro_2_C, Glyco_hydro_2_N	Upregulated
BST61_g6972.t1	Glycoside hydrolase 28	-	Glyco_hydro_28	Upregulated
BST61_g3995.t1	Glycoside hydrolase 29	GH29	Alpha_L_fucos	Upregulated
BST61_g7401.t1	Glycoside hydrolase 3	GH3	Fn3-like, Glyco_hydro_3, Glyco_hydro_3_C, PA14	Upregulated
BST61_g3838.t1	Glycoside hydrolase 3	GH1, GH3	Fn3-like, Glyco_hydro_3, Glyco_hydro_3_C, PA14	Upregulated
BST61_g7876.t1	Glycoside hydrolase 3	GH3	Fn3-like, Glyco_hydro_3, Glyco_hydro_3_C	Upregulated
BST61_g5004.t1	Glycoside hydrolase 3	GH3	Fn3-like, Glyco_hydro_3, Glyco_hydro_3_C	Upregulated
BST61_g9731.t1	Glycoside hydrolase 3	GH3	Fn3-like, Glyco_hydro_3, Glyco_hydro_3_C	Upregulated
BST61_g5668.t1	Glycoside hydrolase 3	GH3	Fn3-like, Glyco_hydro_3, Glyco_hydro_3_C	Upregulated
BST61_g4959.t1	Glycoside hydrolase 3	GH3	Fn3-like, Glyco_hydro_3, Glyco_hydro_3_C	Upregulated
BST61_g4499.t1	Glycoside hydrolase 3 N terminal domain	-	Glyco_hydro_3, Glyco_hydro_3_C	Upregulated
BST61_g9341.t1	Glycoside hydrolase 31	-	Glyco_hydro_31	Upregulated
BST61_g1204.t1	Glycoside hydrolase 31	GH31	Gal_mutarotas_2, Glyco_hydro_31, NtCtMGAM_N	Upregulated
BST61_g1615.t1	Glycoside hydrolase 31	GH31	CBM_20, Gal_mutarotas_2, Glyco_hydro_31, NtCtMGAM_N	Upregulated
BST61_g11216.t1	Glycoside hydrolase 43	-	-	Upregulated
BST61_g6545.t1	Glycoside hydrolase 43	-	Glyco_hydro_43	Upregulated
BST61_g8410.t1	Glycoside hydrolase 43	-	Glyco_hydro_43	Upregulated

BST61_g7505.t1	Glycoside hydrolase 43	-	Glyco_hydro_43	Upregulated
BST61_g5338.t1	Glycoside hydrolase 43	-	Glyco_hydro_43	Upregulated
BST61_g1795.t1	Glycoside hydrolase 43	GH43	Glyco_hydro_43	Upregulated
BST61_g1756.t1	Glycoside hydrolase 43	-	Glyco_hydro_43	Upregulated
BST61_g1844.t1	Glycoside hydrolase 47	GH47	Glyco_hydro_47	Upregulated
BST61_g7848.t1	Glycoside hydrolase 5	GH5, GH9	CBM_1, Cellulase	Upregulated
BST61_g757.t1	Glycoside hydrolase 5 (cellulase A)	-	Cellulase	Upregulated
BST61_g5679.t1	Glycoside hydrolase 5 (cellulase A)	-	Cellulase	Upregulated
BST61_g3230.t1	Glycoside hydrolase 5 (cellulase A)	-	Cellulase	Upregulated
BST61_g11083.t1	Glycoside hydrolase 5 (cellulase A)	GH5, GH9	CBM_1, Cellulase	Upregulated
BST61_g9362.t1	Glycoside hydrolase 51	GH51	Alpha-L-AF_C	Upregulated
BST61_g6966.t1	Glycoside hydrolase 61	-	Glyco_hydro_61	Upregulated
BST61_g714.t1	Glycoside hydrolase 61	AA9, CBM1	Glyco_hydro_61	Upregulated
BST61_g1231.t1	Glycoside hydrolase 62	-	CBM_1, Glyco_hydro_62	Upregulated
BST61_g7175.t1	Glycoside hydrolase 64	-	Glyco_hydro_64	Upregulated
BST61_g10294.t1	Glycoside hydrolase 7 (cellulase C)	GH7	CBM_1, Glyco_hydro_7	Upregulated
BST61_g3467.t1	Glycoside hydrolase 71	-	Glyco_hydro_71	Upregulated
BST61_g3261.t1	Glycoside hydrolase 76	GH76	Glyco_hydro_76	Upregulated
BST61_g10942.t1	Glycoside hydrolase 76	-	Glyco_hydro_76	Upregulated
BST61_g1263.t1	Glycoside hydrolase 78	-	Bac_rhamnosid6H, Bac_rhamnosid_C	Upregulated
BST61_g9809.t1	Glycoside hydrolase 79 C-terminal beta domain	-	Glyco_hydro_79C	Upregulated
BST61_g9655.t1	Glycoside hydrolase 79 C-terminal beta domain	-	Glyco_hydro_79C	Upregulated
BST61_g5716.t1	Glycoside Hydrolase 88	GH105	Glyco_hydro_88	Upregulated
BST61_g11607.t1	Glycoside Hydrolase 88	GH105	Glyco_hydro_88	Upregulated
BST61_g7294.t1	Glycoside hydrolase 92	-	Glyco_hydro_92	Upregulated
BST61_g11404.t1	Glycoside hydrolases 11	-	CBM_1, Glyco_hydro_11	Upregulated
BST61_g2125.t1	Glycoside hydrolases 16	-	Glyco_hydro_16	Upregulated
BST61_g6730.t1	Glycoside hydrolases 28	-	Glyco_hydro_28	Upregulated
BST61_g4274.t1	Glycoside hydrolases 28	-	Glyco_hydro_28	Upregulated
BST61_g7622.t1	Glycoside hydrolases 31	-	Glyco_hydro_31	Upregulated
BST61_g1094.t1	Glycoside hydrolases 43	-	Glyco_hydro_43	Upregulated
BST61_g7157.t1	Glycoside hydrolase 1	GH1	Glyco_hydro_1	Downregulated
BST61_g5823.t1	Glycoside hydrolase 10 (cellulase F)	-	Glyco_hydro_10	Downregulated
BST61_g4820.t1	Glycoside hydrolase 16	GH16	SKN1	Downregulated
BST61_g1225.t1	Glycoside hydrolase 16	GH16	SKN1	Downregulated
BST61_g4869.t1	Glycoside hydrolase 16	-	Glyco_hydro_16	Downregulated
BST61_g2449.t1	Glycoside hydrolase 16	-	Glyco_hydro_16	Downregulated
BST61_g11151.t1	Glycoside hydrolase 16	-	Acyl_transf_3, Glyco_hydro_16	Downregulated
BST61_g6335.t1	Glycoside hydrolase 17	GH17	But2, Glyco_hydro_17	Downregulated
BST61_g1529.t1	Glycoside hydrolase 18	GH18	CBM_19, Flocculin_t3, Glyco_hydro_18	Downregulated
BST61_g9117.t1	Glycoside hydrolase 3	GH3	Fn3-like, Glyco_hydro_3, Glyco_hydro_3_C	Downregulated

BST61_g10220.t1	Glycoside hydrolase 3	GH3	Fn3-like, Glyco_hydro_3, Glyco_hydro_3_C	Downregulated
BST61_g5978.t1	Glycoside hydrolase 32	GH32	Glyco_hydro_32C, Glyco_hydro_32N	Downregulated
BST61_g9676.t1	Glycoside hydrolase 47	GH47	Glyco_hydro_47	Downregulated
BST61_g9186.t1	Glycoside hydrolase 5 (cellulase A)	GH5	Cellulase	Downregulated
BST61_g886.t1	Glycoside hydrolase 5 (cellulase A)	GH5, GH9	Cellulase	Downregulated
BST61_g8572.t1	Glycoside hydrolase 55	-	Pectate_lyase_3	Downregulated
BST61_g5127.t1	glycoside hydrolase 63	-	Glyco_hydro_63	Downregulated
BST61_g6354.t1	Glycoside hydrolase 76	GH76	Glyco_hydro_76	Downregulated
BST61_g8411.t1	Glycoside hydrolase 88	GH105	Glyco_hydro_88	Downregulated
BST61_g10444.t1	Glycoside hydrolase catalytic core	-	Glyco_hydro_cc	Downregulated
BST61_g3550.t1	Glycoside hydrolases 15	GH15	CBM_20, Glyco_hydro_15	Downregulated
BST61_g7199.t1	Glycosyltransferase 1	-	-	Upregulated
BST61_g10964.t1	Glycosyltransferase 25	-	-	Upregulated
BST61_g7402.t1	Glycosyltransferase 25	-	-	Upregulated
BST61_g9763.t1	Glycosyltransferase 25	-	-	Upregulated
BST61_g9765.t1	Glycosyltransferase 25	-	-	Upregulated
BST61_g11001.t1	Glycosyltransferase 31	-	-	Upregulated
BST61_g4445.t1	Glycosyltransferase 31	-	-	Upregulated
BST61_g8953.t1	Glycosyltransferase 31	-	-	Upregulated
BST61_g8966.t1	Glycosyltransferase 31	-	-	Upregulated
BST61_g8440.t1	Glycosyltransferase 32	och1	GT32	Upregulated
BST61_g4662.t1	Glycosyltransferase 34	MNN10	GT34	Upregulated
BST61_g6506.t1	Glycosyltransferase 4	ALG2	GT4	Upregulated
BST61_g4056.t1	Glycosyltransferase 59	ALG10	GT59	Upregulated
BST61_g5577.t1	Glycosyltransferase 8	-	-	Upregulated
BST61_g11062.t1	Glycosyltransferase sugar-binding region containing DXD motif	-	-	Upregulated
BST61_g2486.t1	Glycosyltransferase sugar-binding region containing DXD motif	-	-	Upregulated
BST61_g7365.t1	Glycosyltransferase 1	-	-	Downregulated
BST61_g1026.t1	Glycosyltransferase 2	chsD	GT2	Downregulated
BST61_g3390.t1	Glycosyltransferase 2	CHS3	GT2	Downregulated
BST61_g4223.t1	Glycosyltransferase 2	-	-	Downregulated
BST61_g4950.t1	Glycosyltransferase 25	-	-	Downregulated
BST61_g2287.t1	Glycosyltransferase 48	FKS1	GT48	Downregulated

[1]Description assigned by eggnog-mapper v2 based on orthology mapping.
[2]Hits against the carbohydrate-active enzymes (CAZy) database.
[3]Hits against the PFAM database

Table S4. Predicted cellular localisation of *Cercospora zeina* candidate secreted effector proteins. Effector localisation was predicted using SignalP6.0, EffectorP 3.0-fungi and localizer.

**Localization**	**Total**	**[1]Differentially expressed**
Apoplastic	173	95
[2]Apoplastic/Cytoplasmic	25	11
Cytoplasmic	105	30
[3]Cytoplasmic/Apoplastic	8	4
[4]mTP	2	0
[5]cTP	10	3
[6]NLS	28	7

[1]Protein products of genes showing differential expression in at least one contrast.
[2]Predicted to be dual localized, with apoplastic localization favoured.
[3]Predicted to be dual localized, with cytoplasmic localization favoured.
[4]mTP – mitochondrial transit peptide.
[5]cTP – Chloroplast transit peptide.
[6]NLS – Nuclear localization signal.

Table S5. BLASTp hit table using the C-terminal 122 aminos of *Cercospora zeina* NIS1b as the search query

Description	Max Score	Total Score	Query Cover	E value	Per. ident	Amino acid Acc. Len	Accession
unnamed protein product [Cercospora beticola]	305	305	100%	4.00E-102	94.94	299	CAK1358316.1
uncharacterized protein RHO25_007619 [Cercospora beticola]	258	258	100%	2.00E-83	94.94	299	XP_023448738.1
uncharacterized protein CKM354_000478100 [Cercospora kikuchii]	256	256	100%	2.00E-82	94.3	299	XP_044655965.1
hypothetical protein CBER1_06471 [Cercospora berteroae]	255	255	100%	3.00E-82	93.04	299	PPJ50310.1
hypothetical protein Slin14017_G064900 [Septoria linicola]	200	200	98%	2.00E-60	85.16	299	KAI5362809.1
hypothetical protein TI39_contig297g00027 [Zymoseptoria brevis]	149	149	100%	1.00E-40	55	273	KJY01316.1
unnamed protein product [Zymoseptoria tritici ST99CH_1E4]	142	142	81%	4.00E-38	60	289	SMR58604.1
uncharacterized protein M409DRAFT_27203 [Zasmidium cellare ATCC 36951]	142	142	100%	5.00E-38	57.41	296	XP_033663471.1
unnamed protein product [Zymoseptoria tritici ST99CH_3D7]	141	141	81%	1.00E-37	60	289	SMQ54170.1
signal peptide-containing protein [Zymoseptoria tritici IPO323]	136	136	81%	1.00E-35	57.36	288	XP_003849443.1
hypothetical protein PRZ48_013904 [Zasmidium cellare]	131	131	79%	9.00E-34	61.9	295	KAK4494548.1
uncharacterized protein RCC_10736 [Ramularia collo-cygni]	129	129	78%	7.00E-33	51.22	278	XP_023631731.1
Hypothetical predicted protein [Lecanosticta acicola]	127	127	84%	3.00E-32	49.26	290	CAK4031412.1
